# Mutagenetic analysis of the biosynthetic pathway of tetramate bripiodionen bearing 3-(2*H*-pyran-2-ylidene)pyrrolidine-2,4-dione skeleton

**DOI:** 10.1186/s12934-024-02364-7

**Published:** 2024-03-21

**Authors:** Haixia Zang, Yijia Cheng, Mengjia Li, Lin Zhou, Li-Li Hong, Hai Deng, Hou-Wen Lin, Yongjun Zhou

**Affiliations:** 1 Research Center for Marine Drugs, Department of Pharmacy, Ren Ji Hospital, School of Medicine, Shanghai Jiao Tong University, Shanghai, 200127 China; 2 Department of Chemistry, University of Aberdeen, Aberdeen, AB24 3UE UK

**Keywords:** Tetramic acid, Dieckmann cyclase, Nonribosomal peptide synthetase, Aminoacyl transthiolation

## Abstract

**Background:**

Natural tetramates are a family of hybrid polyketides bearing tetramic acid (pyrrolidine-2,4-dione) moiety exhibiting a broad range of bioactivities. Biosynthesis of tetramates in microorganisms is normally directed by hybrid polyketide synthase (PKS) and nonribosomal peptide synthetase (NRPS) machineries, which form the tetramic acid ring by recruiting *trans*- or *cis*-acting thioesterase-like Dieckmann cyclase in bacteria. There are a group of tetramates with unique skeleton of 3-(2*H*-pyran-2-ylidene)pyrrolidine-2,4-dione, which remain to be investigated for their biosynthetic logics.

**Results:**

Herein, the tetramate type compounds bripiodionen (BPD) and its new analog, featuring the rare skeleton of 3-(2*H*-pyran-2-ylidene)pyrrolidine-2,4-dione, were discovered from the sponge symbiotic bacterial *Streptomyces reniochalinae* LHW50302. Gene deletion and mutant complementation revealed the production of BPDs being correlated with a PKS-NRPS biosynthetic gene cluster (BGC), in which a Dieckmann cyclase gene *bpd*E was identified by sit-directed mutations. According to bioinformatic analysis, the tetramic acid moiety of BPDs should be formed on an atypical NRPS module constituted by two discrete proteins, including the C (condensation)-A (adenylation)-T (thiolation) domains of BpdC and the A-T domains of BpdD. Further site-directed mutagenetic analysis confirmed the natural silence of the A domain in BpdC and the functional necessities of the two T domains, therefore suggesting that an unusual aminoacyl transthiolation should occur between the T domains of two NRPS subunits. Additionally, characterization of a LuxR type regulator gene led to seven- to eight-fold increasement of BPDs production. The study presents the first biosynthesis case of the natural molecule with 3-(2*H*-pyran-2-ylidene)pyrrolidine-2,4-dione skeleton. Genomic mining using BpdD as probe reveals that the aminoacyl transthiolation between separate NRPS subunits should occur in a certain population of NRPSs in nature.

**Supplementary Information:**

The online version contains supplementary material available at 10.1186/s12934-024-02364-7.

## Introduction

**Fig. 1:**
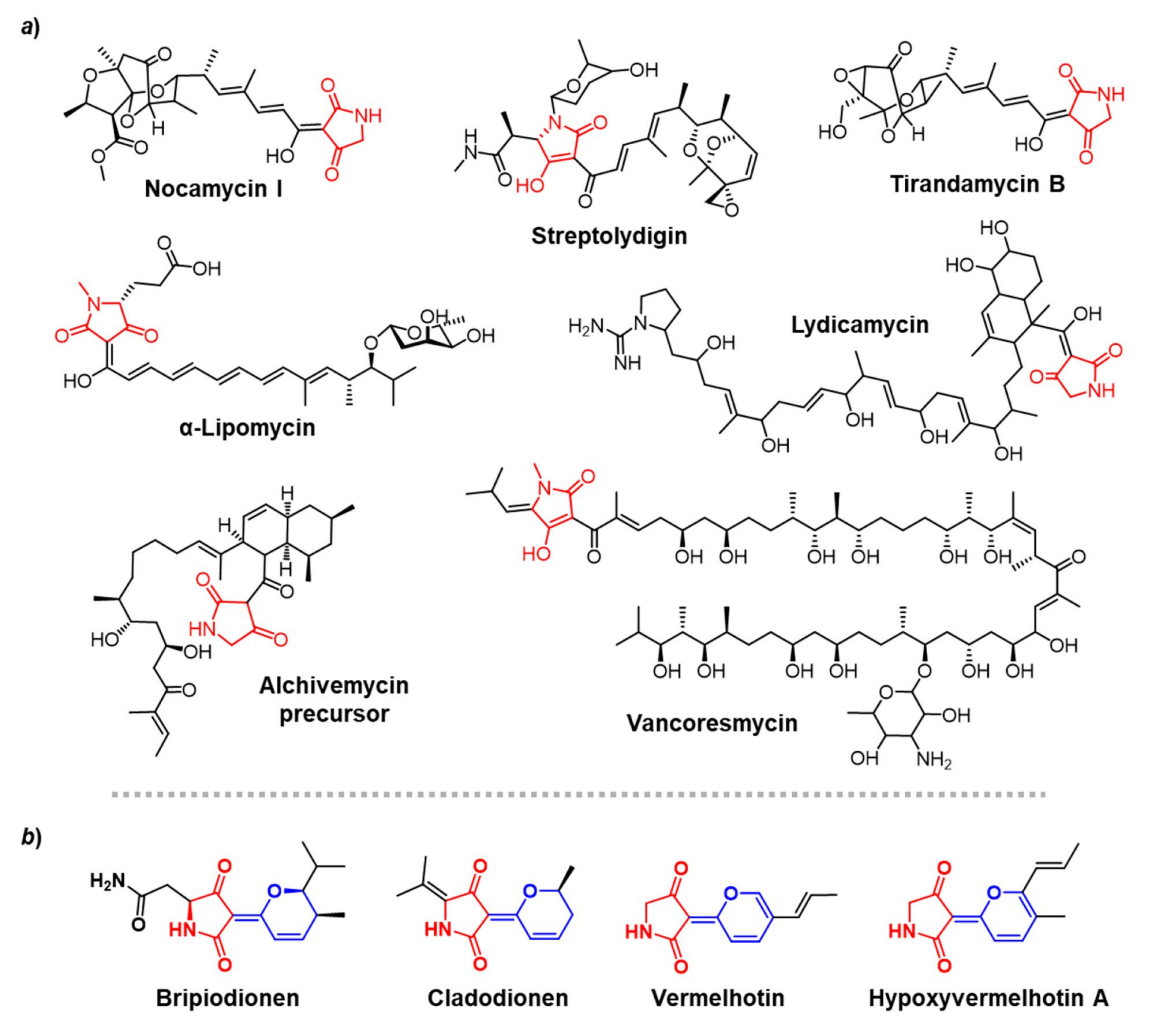
The tetramates derived from the biosynthetic pathways that involve discrete TE-like Dieckmann cyclases (***a***) and the tetramates bearing 3-(2*H*-pyran-2-ylidene)pyrrolidine-2,4-dione skeleton (marked in red and blue) (***b***)

Natural tetramates, structurally featured with a tetramic acid (pyrrolidine-2,4-dione) moiety, are discovered from diverse organism resources exhibiting a broad range of bioactivities such as antibiotic, antitumor, antifungal and antiviral [1–3]. The biosynthesis of tetramates is generally directed by hybrid multimodular polyketide synthase (PKS) and nonribosomal peptide synthetases (NRPS) machineries, in which the tetramic acid moiety is formed at the terminal NRPS module via a Dieckmann cyclization [4]. The Dieckmann cyclizations occurred in bacterial tetramates biosynthetic pathways are normally catalyzed by atypical C-terminal thioesterase (TEs) domains or free-standing TE-like enzymes [4]. The genes of free-standing TE-like enzymes were discovered in the BGCs of a group of tetramates such as tirandamycin B (*trd*C) [5], streptolydigin (*slg*L) [6], α-lipomycin (*lip*X2) [7], lydicamycin (*TPA0598*_03_00810) [8], vancoresmycin (*var*15) [9], nocamycin I (*ncm*C) [10], and alchivemycin (*avm*Q) (Fig. 1a) [11]. Among the proteins of these genes, the Dieckmann cyclase activities of TrdC, SlgL, LipX2, and NcmC were evidenced by biochemical analysis with the *N*-acetoacetyl substrate mimics [12, 13]. According to the crystal structure of NcmC [13], this type of Dieckmann cyclases harbor an *α*/*ß* hydrolase fold subdomain and a four-helical bundle subdomain. The former resembles the canonical TE domain with a slightly altered catalytic triad of Cys–Asp–His compared to the Ser–Asp–His in TEs. The latter represents the unique structure of this class of Dieckmann cyclases, being proposed to participate in polyketide tail recognition [13].

As one of the key elements for tetramates biosynthesis, the NRPS module is basically composed of an adenylation (A) domain, which activates specific amino acid building block as an aminoacyl adenylate, a thiolation (T) domain, which tethers the amino acid through a 4′-phosphopantetheine (Ppant) linker, and a condensation (C) domain, which catalyzes peptide bond formation [14]. Interestingly, some NRPS assembly lines deviate from the canonical C-A-T modular architecture by recruiting the “A-less” C-T modules, which receive amino acid units from standalone A-T didomain modules by the mediation of aminoacyl-shuttling enzymes such as the atypical type II TEs from the biosynthetic pathways of cyclodepsipeptide WS9326A and pyrrolizidine alkaloid legonindolizidines [15, 16], and the aminoacyltransferase from lipopeptidolactone syringomycin biosynthetic pathway [17].

Of the natural tetramates, a family produced by microorganisms, featuring the rare 3-(2*H*-pyran-2-ylidene)pyrrolidine-2,4-dione skeleton, remain unknown for their biosynthetic logics such as bripiodionen (BPD) from bacteria with inhibitory activity against human cytomegalovirus protease [18] and the three ones from fungus including cladodionen with cytotoxic activities against human cancer cells [19], vermelhotin with antiplasmodial activity [20], and hypoxyvermelhotin A with cytotoxicity against murine fibroblast cell line L-929 (Fig. 1b) [21]. In this study, a new analog of BPD is discovered in a sponge symbiotic *Streptomyces* strain. Mutagenetic analysis of the *bpd* BGC suggests that the BPDs biosynthesis involved an unusual aminoacyl transthiolation between separate NRPS subunits and a Dieckmann cyclization. The production of BPDs was also improved significantly by manipulating a regulator gene. The study presents the first biosynthesis case for 3-(2*H*-pyran-2-ylidene)pyrrolidine-2,4-dione skeleton, facilitating further structural diversification through bioengineering.

## Results and disscussion

### Discovery of BPDs from the fermentation of LHW50302

**Fig. 2:**
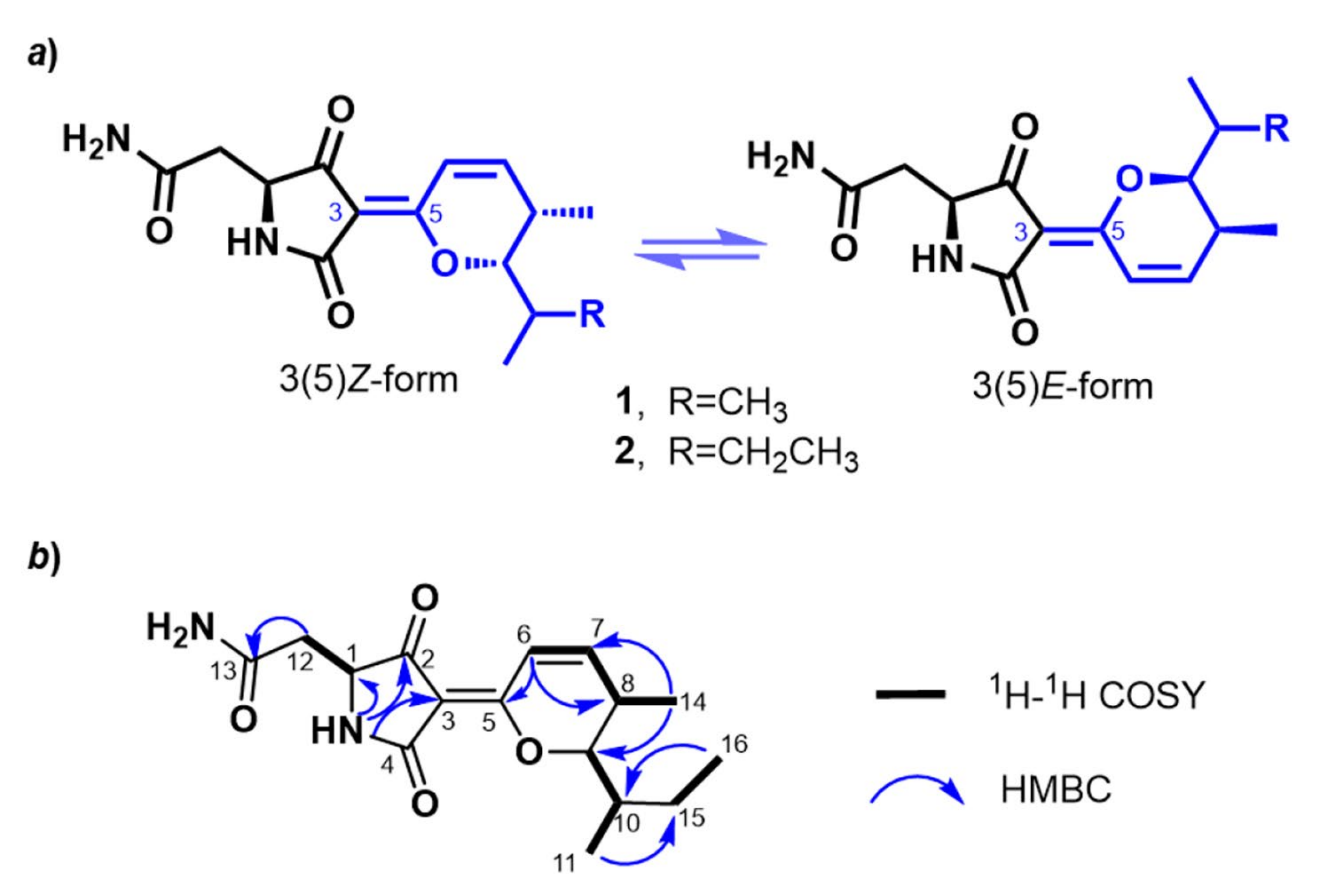
Structures of compounds **1** and **2** (***a***), and the ^1^H-^1^H COSY and HMBC correlations of **2** (***b***)

Sponge ecosystem contains rich and divers microorganisms which act as significant origins of the medicinal active molecules discovered from sponge [22, 23]. During secondary metabolite screening of the sponge symbiotic *Streptomyces reniochalinae* LHW50302 [24], two tetramates, including the known bripiodionen (BPD) (**1**) and a new BPD analog, BPD B (**2**), were discovered (Fig. 2a). Compound **1**, obtained as a white powder, presented as a mixture of inseparable *E*/*Z*-Δ^3(5)^ geometric isomers (**1a** and **1b**). The molecular formula C_15_H_20_N_2_O_4_ was determined by the HRESIMS at *m*/*z* 293.1502 [M + H]^+^ (calcd 293.1496), accounting for seven degrees of unsaturation. The ^1^H and ^13^C NMR spectra of **1** are identified to those of BPD discovered from *Streptomyces* sp. WC76599 [18] (Table S3), which have two sets of resonances with a ratio of 1:3 for **1a** and **1b** (Fig. S6).

Compound **2**, isolated as a white powder, had the molecular formula C_16_H_22_N_2_O_4_ according to the HRESIMS at *m*/*z* 307.1649 [M + H]^+^ (calcd 307.1652), with seven degrees of unsaturation. Similar to compound **1**, compound **2** was obtained as a mixture of two inseparable geometric isomers **2a** (*E*-Δ^3(5)^) and **2b** (*Z*-Δ^3(5)^) in a 1:3 ratio (Fig. S7). The ^1^H and ^13^C NMR data of **2** were almost identical to those of **1** with the exception that the methyl at C-10 in **1** (*δ*_C_ 18.8/19.2, CH_3_*-*15) (Table S3) was replaced with the ethyl in **2** (*δ*_C_ 23.8/23.9, CH_2_*-*15; *δ*_C_ 13.4/13.1, CH_3_*-*16) (Fig. 2, Table S4). This assignment was further supported by the COSY correlation between H_2_-15 and H_3_-16, and the HMBC correlation from H_3_-16 to C-10 (Fig. 2b, Table S4). The NMR data of **2a** and **2b** were nearly identical with the exception of chemical shift at H-6 (*δ*_H−6_ 7.46/7.62) for **2a**/**2b** (Table S4), which could be caused by the deshielding effect of C-2 carbonyl.

### Identification of BPDs biosynthetic gene cluster in LHW50302

**Fig. 3:**
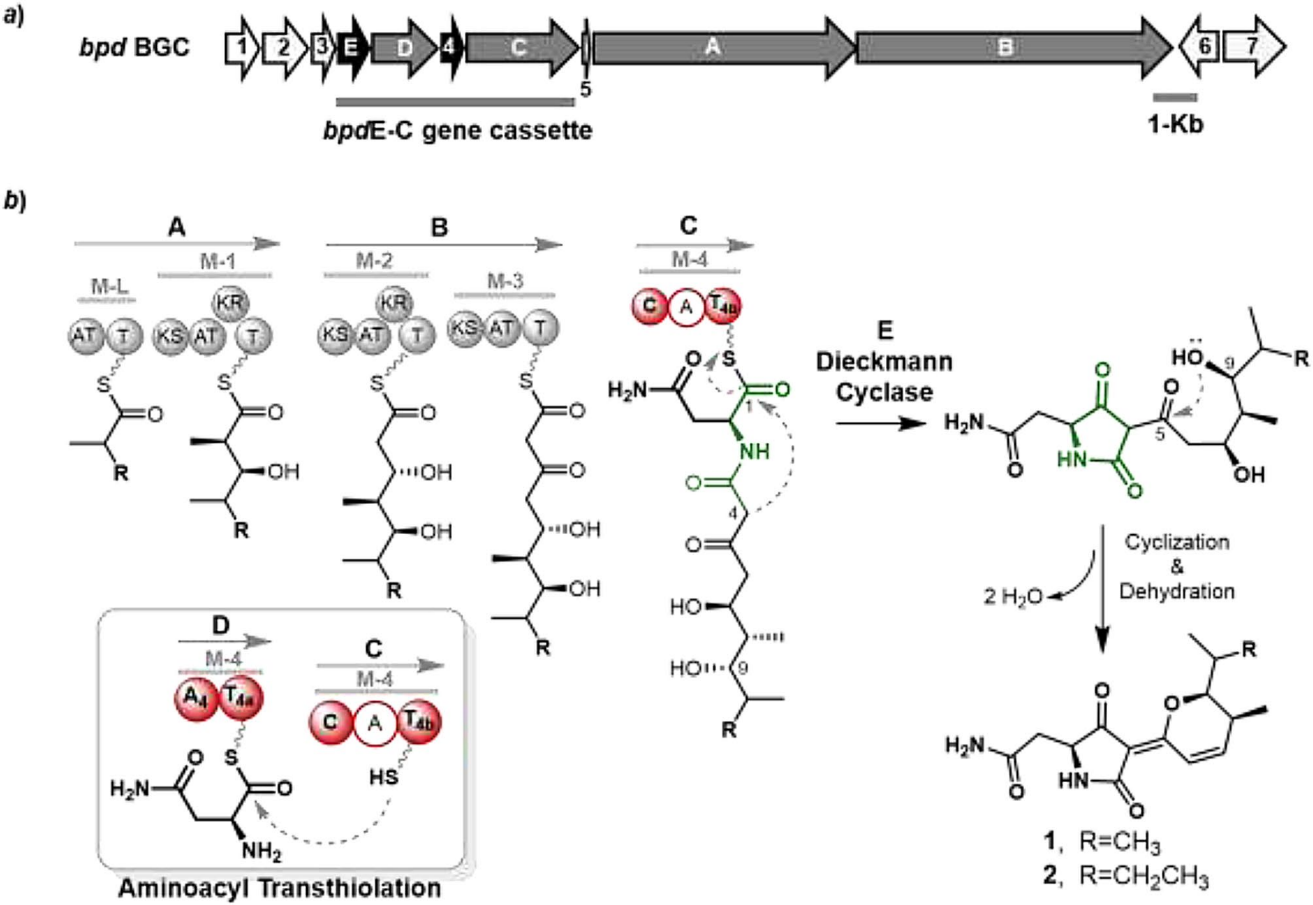
Gene organization of *bpd* BGC (***a***) and the proposed biosynthetic pathway of compounds **1** and **2** (***b***). The proposed aminoacyl transthiolation between the BpdD and BpdC NRPS subunits is highlighted by a frame; The inactive A domain in BpdC is shown by white circle

Natural tetramate molecules are generally constructed from polyketide and *α*-amino acid units via the concerted actions of PKS and NRPS assemblies. In bacteria, the tetramate scaffold is usually formed from a nascent linear product tethered on NRPS module via the Dieckmann cyclization catalyzed by an atypical C-terminal thioesterase (TE) domain or free-standing TE-like enzyme [4]. With the information, BLAST search against LHW50302 genome was performed using TrdC as the query seed, which was identified as a Dieckmann cyclase in the biosynthetic pathway of tirandamycin from *Streptomyces* sp. SCSIO 1666 [12]. The analysis led to discovery of an open reading frame (ORF), named as *bpd*E, showing 36% protein sequence identity to TrdC. The *bpd*E gene is located within an unknown PKS-NRPS BGC, termed as *bpd* BGC (Fig. 3a), which basically harbors two PKS genes *bpd*A and *bpd*B and two NRPSs genes *bpd*C and *bpd*D (Table 1). Based on antiSMASH analysis [25] and the structural features of BPDs, the PKS-NRPS assembly line of BPDs should be initiated with the starting unit of isobutyryl-CoA for compound **1** or 2-methylbutyryl-CoA for compound **2** and then proceeded by condensations of one methymalonyl-CoA, two malonyl-CoAs, and one Asn to yield a nascent hybrid polyketide chain, which could first receive the BpdE-catalyzed heterocyclization to yield the pyrrolidine-2,4-dione core and then undergo a spontaneous pyran-ring formation (Fig. 3b).

**Table 1:** Deduced functions of the ORFs in bpd BGC

Gene	Size (aa)	Proposed function	Homolog (accession No.)	Identity/coverage
**A**	2212	Type I PKS (AT-ACP, KS-AT-KR-ACP)	LipPks1 (ABB05102.1)	53/95
**B**	2629	Type I PKS (KS-AT-KR-ACP, KS-AT- ACP)	CmiP5 (BAO66539.1)	56/99
**C**	900	NRPS (C-A-T)	LipNrps (ABB05101.1)	43/74
**D**	587	NRPS (A-T)	WS23 (QBA57741.1)	52/98
**E**	267	Dieckmann cyclase	NcmC (ARS01469.1)	38/95
**1**	258	Type II thioesterase	LipTe (ABB05106.1)	56/95
**2**	348	LmbU family transcriptional regulator	LmbU (ABX00623.1)	46/55
**3**	162	YbaK/prolyl-tRNAsynthetase	DsaC (UNF16846.1)	50/93
**4**	184	LuxR family transcriptional regulator	HTH_LUXR (KPC91000.1)	79/100
**5**	71	MbtH-like_protein	MbtH (P9WIP5.1)	48/78
**6**	285	XRE family transcriptional regulator	CltP (ACS50121.1)	36/95
**7**	488	Actinorhodin transporter	ActII-2 (P46105.1)	38/91

**Fig. 4:**
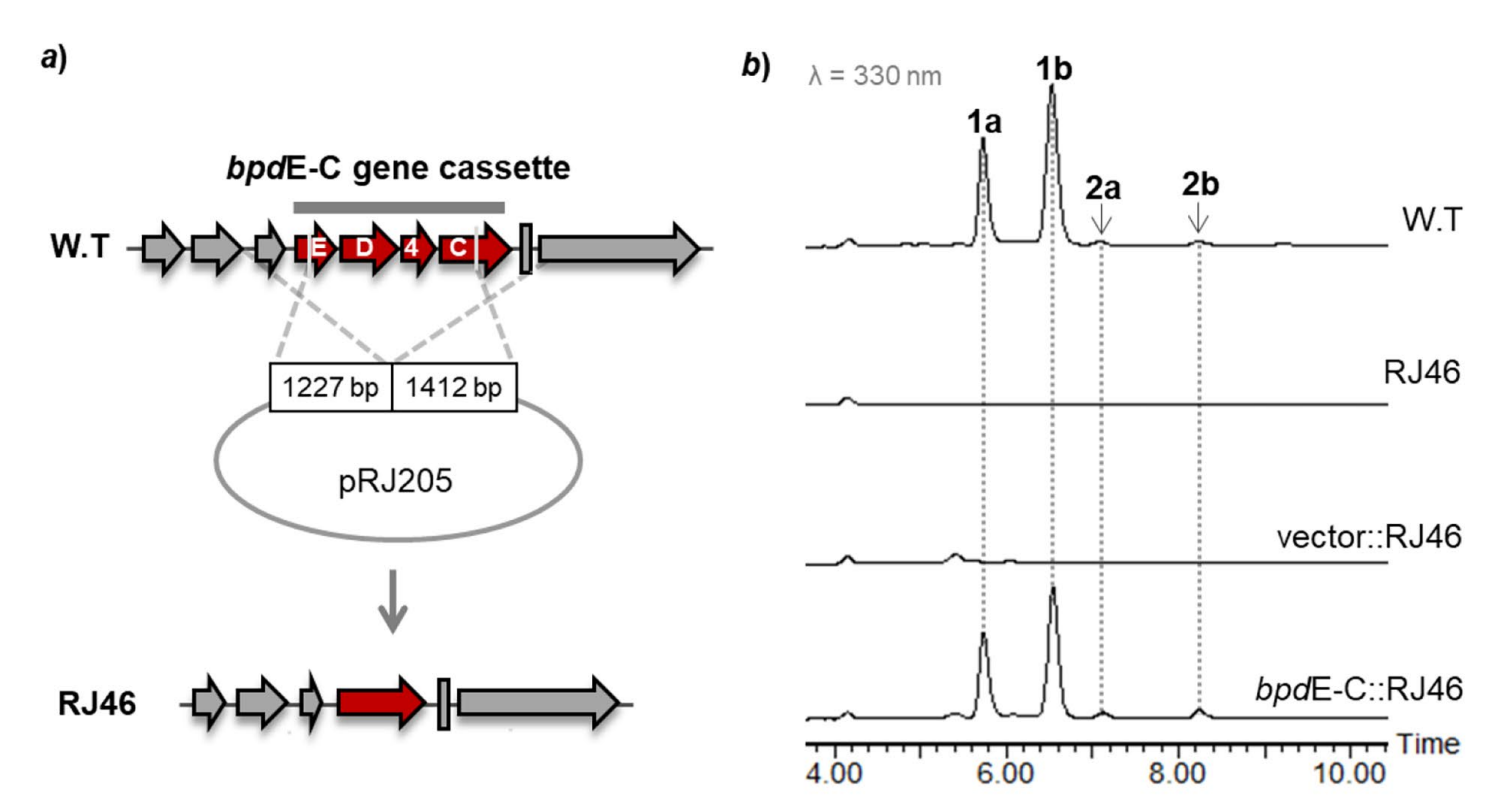
Identification of *bpd* BGC in LHW50302. ***a***) Deletion of *bpd*E-C gene cassette via double-crossover homologous recombination; ***b***) HPLC analysis of compounds **1** and **2** from the resultant mutant and gene complementation strain

To identify the correlation of *bpd* BGC and compounds **1** and **2**, the gene cassette *bpd*E-C consisting of *bpd*C, *bpd*D, *bpd*E and *orf*4 was deleted from the chromosome of LHW50302 via homologous recombination (Fig. 4a). As expected in HPLC analysis of the fermentation extracts (Fig. 4b), the resulting mutant Δ*bpd*E-C indeed lost the production of **1** and **2**, which was then restored when the *bpd*E-C cassette was delivered back into Δ*bpd*E-C via the plasmid pRJ453 constructed from an integrative vector. The results therefore confirmed that the production of **1** and **2** was correlated to *bpd* BGC.

### Identification of the Dieckmann cyclase gene ***bpdE*** by site-directed mutagenetic analysis

**Fig. 5:**
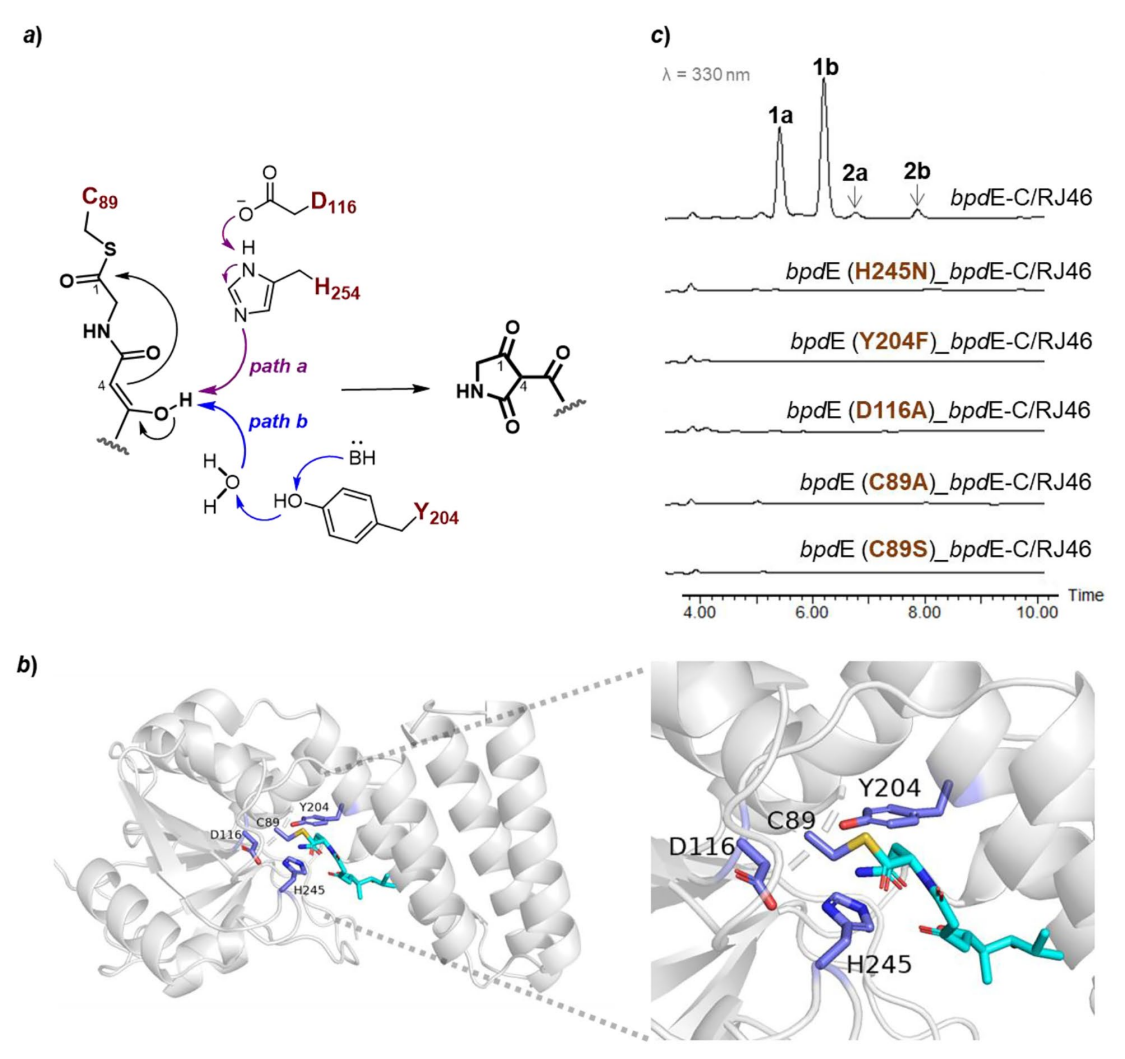
Identification of the Dieckmann cyclase gene *bpd*E by site-directed mutagenetic analysis. ***a***) The documented proposal for the enzymatic mechanism of TE-like Dieckmann cyclases, in which the key active sites are labeled according to BpdE. ***b***) The modeled protein structure of BpdE docked with a linear BPD precursor chain tethered in the Cys_89_ site of ligand binding pocket. ***c***) HPLC analysis of compounds **1** and **2** from the RJ46 (Δ*bpd*E-C) strain complemented with the modified *bpd*E-C gene cassettes bearing sit-directed mutations in *bpd*E

Owing to lack of C-terminal TE domain, the tetramic acid core in BPDs should apparently be formed by a trans-working chain offloading cyclase, for instance the BpdE homologous to TrdC-type Dieckmann cyclase [4]. According to DALI search against the Protein Data Bank (PDB), the BpdE protein structure modeled by AlphaFold2 is most similar (2.1 Å RMSD for 253 Cα atoms) to NcmC (PDB 6E6Y) (Fig. S1), which was biochemically characterized as an offloading Dieckmann cyclase installing a tetramate headgroup in the hybrid polyketide/nonribosomal-peptide nocamycin from *Saccharothrix syringae* [13]. The canonical motif Cys-Asp-His in TrdC-type enzymes is recognized in BpdE by multiple sequence alignment of BpdE with the discrete TE-like Dieckmann cyclases from tetramate biosynthetic pathways (Fig. S2). According to enzymatic mechanism of TE-like Dieckmann cyclases proposed according to protein structure and site-mutation analysis of NcmC [13], the linear backbone of BPDs should be transthiolated from the *HS*-Ppant arm of T_4b_ domain to the Cys_89_ site of BpdE; a nucleophilic cyclization from C4 to C1 is then initiated by the substrate C4 enol, which is activated by the catalytic base of His_245_ stabilized by Asp_116_ or by the proton-shuttle model of Tyr_204_ and one water (Fig. 5a).

To identify the four active sites in BpdE (Fig. 5b), the site mutations of C89A, H245N, D116A, and Y204F were generated based on the *bpd*E-C gene cassette in the plasmid pRJ453, which was successfully used for complementation of the Δ*bpd*E-C mutant strain (Fig. 4b). As expected, the four resultant pRJ453 variants gave no complementary effects to the Δ*bpd*E-C mutant (Fig. 5c), compared to the significant production recovery of compounds **1** and **2** upon introduction of pRJ453, therefore confirming their essential roles for the catalytic activity of BpdE. Moreover, the mutation C89S was also generated to test whether BpdE can employ Ser to anchor linear intermediate by oxoester bound, instead of the thioester bound of Cys active site. The mutation is plausible due to that the Ser–Asp–His catalytic triad is also discovered in the C-terminal TE domains possessing Dieckmann cyclase activities in some bacterial tetramate biosynthetic pathways [4]. Interestingly, the pRJ453 variant containing C89S mutation failed to restore the production of compounds **1** and **2** in Δ*bpd*E-C (Fig. 5c), suggesting different catalytic mechanisms remained to be elucidated in two types of Dieckmann cyclases.

### Site-directed mutagenetic analysis of the A and T domains within the atypical NRPS module encoded by ***bpdC*** and ***bpdD***

According to the structures of BPDs, one Asn elongation unit should be incorporated at the end of the PKS-NRPS assembly line of BPDs. Interestingly, the expected C-terminal NRPS module seemed to be consisted by two discrete proteins BpdC and BpdD, in which BpdC contains C-A-T domains and BpdD is an A-T didomain (Fig. 3). Detail analysis suggested that the A domain in BpdC should be inactive due to the naturally occurred mutations of D552P and K792R (Fig. S3a), which are located in the conservative Asp and Lys sites essential for the catalysis of A domain [26]. Thus, the adenylation of Asn and subsequent aminoacyl thiolation could be carried out by the A domain of BpdD, which contains the Asn specificity-conferring code according to the prediction from antiSMASH. Moreover, both BpdC and BpdD contain bioinformatically intact T domains, T_4a_ and T_4b_ (Fig. S3b), therefore raising a question whether each of them was required for the NRPS module.

**Fig. 6:**
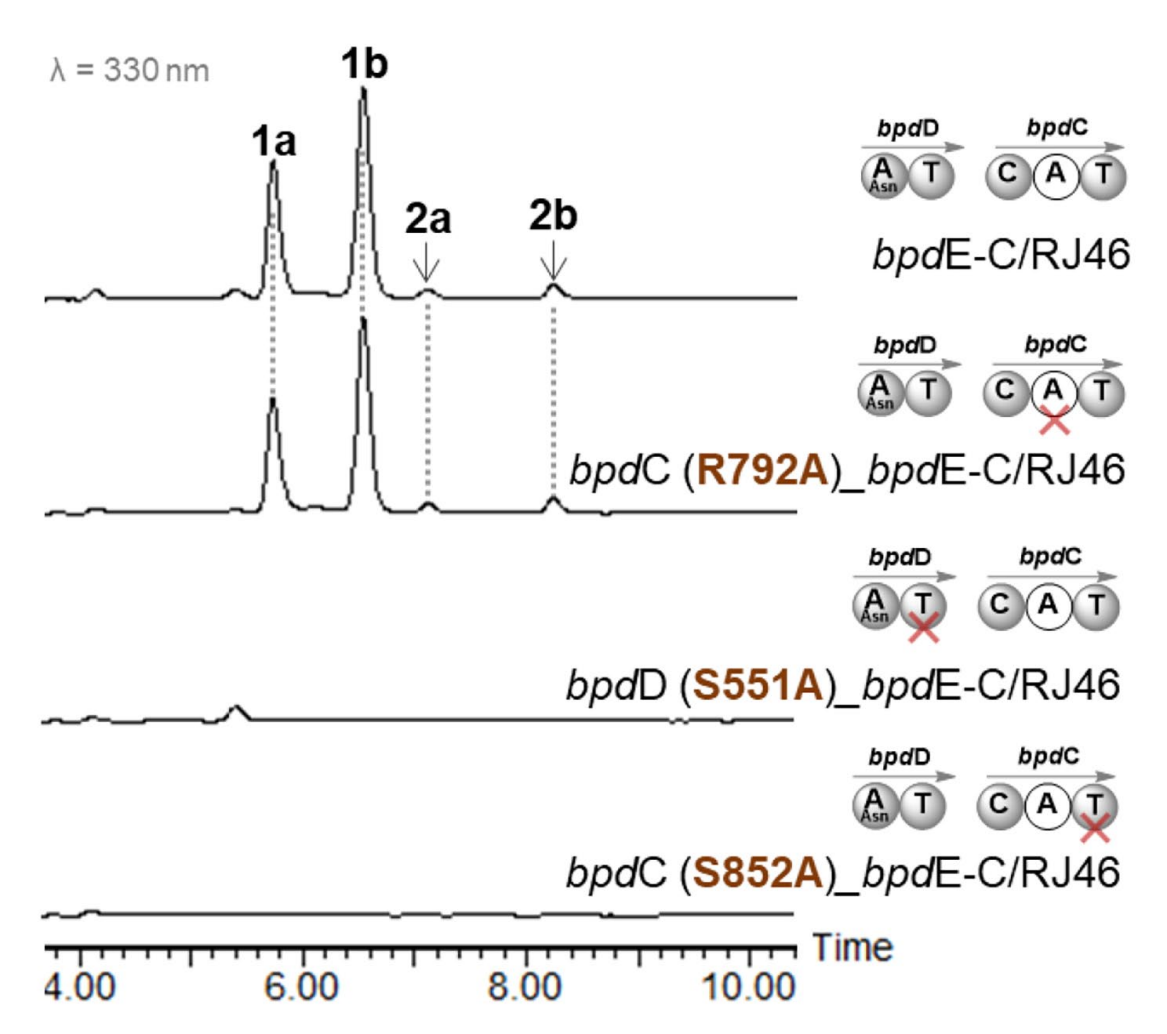
HPLC analysis of compounds **1** and **2** from the RJ46 (Δ*bpd*E-C) strain complemented with the modified *bpd*E-C gene cassette bearing the sit-mutations R792A in *bpd*C, S551A in *bpd*D, and S852A in *bpd*C, respectively. The domains introduced with target site-mutations are indicated by crosses

To address the point, the A domain from *bpd*C and the two T domains from *bpd*D and *bpd*C were individually examined by site-directed mutagenetic analysis. For the investigation, the pRJ453 containing *bpd*E-C genes cassette was used as parent plasmid to generate these mutations. To convincingly inactivate the A domain in BpdC, the R792A mutation in *bpd*C was generated targeting the canonical Arginine active site of A domain [26]. To inactivate the T_4a_ and T_4b_ domains, the S852A mutation in *bpd*C and S551A in *bpd*D were generated to abolish the Serine active site covalently bonded to the Ppant cofactor [27]. These pRJ453 variants were individually introduced into the Δ*bpd*E-C mutant strain and the effects of site-mutations were evaluated by determining the production of compound **1** from the recombinant strains. According to HPLC analysis, the R792A mutation in *bpd*C did not influence the restored production of **1**, whereas each of S551A in *bpd*D and S852A in *bpd*C led to abolished production of **1** (Fig. 6). These results therefore confirm that the A domain in BpdC is naturally inactive and the NRPS module in BPDs biosynthesis should work with two independent T domains.

### Unusual aminoacyl transthiolation in the atypical NRPS module of BPDs biosynthesis

As a speculation based on above mutagenetic analysis of the NRPS domains, incorporation of Asn unit should be performed by an atypical NRPS module, in which the A-T_4a_ didomain of BpdD activates and loads an Asn and then the Asn aminoacyl group is shuttled from T_4a_ domain to the *HS*-Ppant arms of T_4b_ domain in BpdC. The C domain of BpdC then catalyzed the aminoacyl condensation based on T_4b_ domain (Fig. 3b). The aminoacyl group transthiolation between two separate NRPS subunits were observed in rare cases of bacterial secondary metabolites biosynthesis. So far, the documented cases include the aminoacyltransferases CmaE and SyrC from the biosynthetic pathways of cyclopropyl amino acid coronamic acid and lipopeptidolactone syringomycin [17, 28] and the atypical type II TEs WS5/WS20 and LgnA from the biosynthetic pathways of cyclodepsipeptide WS9326A [15] and pyrrolizidine alkaloid legonindolizidines [16].

Given the speculation, the TEII homologous gene *bpd*1 at left side of *bpd* BGC (Fig. 3a), showing 36% identity to LgnA, was investigated by gene deletion. However, the production of **1** and **2** was not affected in the fermentation extract of resultant Δ*bpd*1 mutant strain according to HPLC–MS analysis (Fig. S4). Moreover, BLAST search using SyrC as query seed did not reveal obvious aminoacyltransferase homologous genes from the genome of LHW50302. As an alternative mechanism, the A-T didomain of BpdD can independently carry out the aminoacyl transthiolation between two T domains since this type of A-T didomain genes are present in a certain population of NRPS BGCs according to the survey of documented BGCs of NRPSs (Fig. S5), though their evolutional significance remined to be investigated.

### Boosting BPDs production by enhancing the transcription level of LuxR type regulator gene

**Fig. 7:**
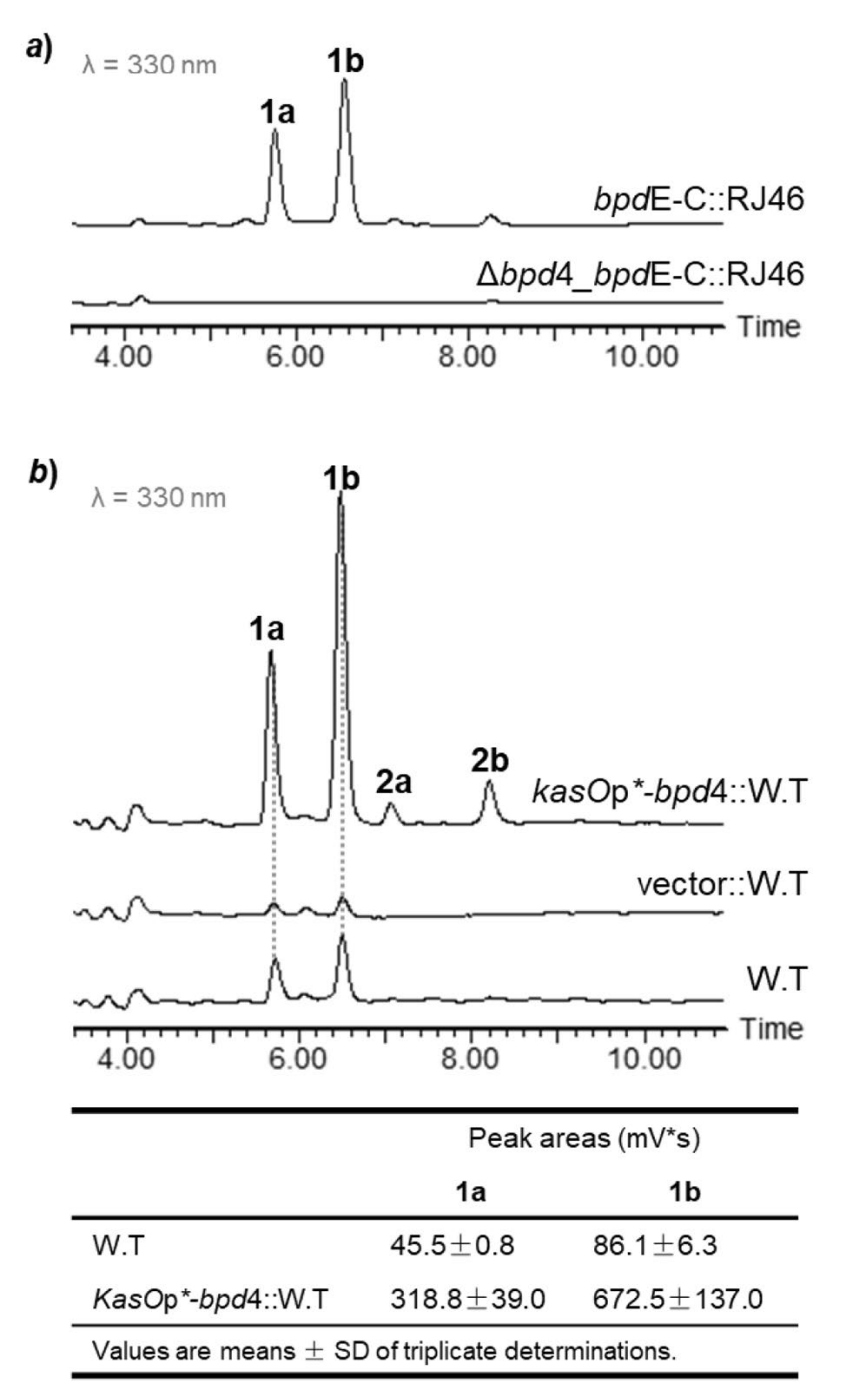
Improving BPD production by overexpressing the regulator gene *bpd*4. ***a***) HPLC analysis of compound **1** from the RJ46 (Δ*bpd*E-C) strain complemented with a *bpd*E-C cassette derivative (Δ*bpd*4*_bpd*E-C) with the regulator gene *bpd*4 deleted. ***b***) HPLC analysis of compound **1** from the LHW50302 (W.T) introduced with extra copy of *bpd*4 gene engaged with a constitutive and strong promoter *kasO*p*. The production improvement levels of **1a** and **1b** were evaluated by calculating the target peak areas shown in the table at bottom

Protein sequence analysis suggests the product of *bpd*4 gene belonging to the LuxR family transcription regulators, which are characterized by the C-terminal helix-turn-helix (HTH) domain [29, 30]. To functionally identify *bpd*4, the ORF of *bpd*4 was deleted from the *bpd*E-C cassette in pRJ453, and when the resultant plasmid was introduced into Δ*bpd*E-C strain, the production of compounds **1** and **2** was totally destroyed (Fig. 7a), suggesting Bpd4 as a strict regulator in BPDs biosynthesis. The result encouraged us to boost BPDs production by overexpression of *bpd*4 gene based on an integrative vector in LHW50302 strain. The ORF of *bpd*4 gene was then engaged with a strong and constitutive promoter *kasO*p*, which was demonstrated with good compatibilities in a broad array of *Actinomyces* hosts [31]. As expected, seven- to eight-fold yield improvement of compound **1** was accomplished upon overexpression of *bpd*4 in LHW50302 according to HPLC analysis of the fermentation extracts (Fig. 7b).

## Concluding remarks

Two tetramate type compounds, including the known BPD and a new BPD analog, featuring the rare skeleton of 3-(2*H*-pyran-2-ylidene)pyrrolidine-2,4-dione, were discovered from sponge symbiotic *Streptomyces reniochalinae* LHW50302. Gene deletion and mutant complementation demonstrated that the production of BPDs was directed by a PKS-NRPS type BGC, in which a Dieckmann cyclase gene *bpd*E was identified by sit-directed mutations. Site-directed mutagenetic analysis of the NRPS domains suggests that the tetramic acid moiety of BPDs should be formed on an atypical NRPS module constituted by two separate subunits, in which an aminoacyl transthiolation should occur between two T domains. In addition, characterization of a LuxR type positive regulator gene led to seven- to eight-fold increasement of the BPD production. The study presents the first biosynthetic pathway of the natural product containing 3-(2*H*-pyran-2-ylidene)pyrrolidine-2,4-dione skeleton. Moreover, genomic mining using BpdD as probe reveals a certain population of identified NRPSs containing both freestanding A-T didomain and A-less module, implying the generality of this type of aminoacyl transthiolation between NRPS subunits in nature.

## Materials and methods

### DNA manipulation and chemicals

Primers synthesis and DNA sequencing were ordered from Shanghai Sangon Biotech (China). DNA fragments were assembled by using 2 × Ezmax-Muli CloneMix Plus (Shanghai Tolo Biotech, China). PCR amplifications were carried out by using KOD OnePCR Master Mix (TOYOBO) for gene cloning and by using 2 × FastTaq Master Mix (Shanghai Bioroot Biotech) for colony screening. Plasmid DNA extraction was performed by using plasmid mini kit (Shanghai Generay Biotech). The genomic DNA used as a PCR template was prepared by using 10% Chelex 100 resin (Bio-Rad) solution.

### Bactetial strains, culture conditions, and plasmids

*E. coli* DH10B was used as a host for plasmid construction. *E.coli* ET12567 strain containing pUZ8002 plasmid was used for introducing plasmid into *Streptomyces*. The sponge symbiotic *S. reniochalinae* LHW50302 [24] was used as the starting strain to investigate *bpd* BGC. *E. coli* strains were grown at 37 °C in Luria-Bertani (LB) broth (1% tryptone, 0.5% yeast extract, 0.5% NaCl) or the solid LB medium (plus 1.5% agar) supplemented with corresponding antibiotics. SFM agar medium (2% soyean flour, 2% D-mannitol, 2% agar, pH 7.2) was used for conjugation and mutant screening. For *Streptomyces* fermentation, the TSBY medium (3% tryptone soy broth, 0.5% yeast extract, 10% sucrose, 0.1% antifoam) was used for seed culture and the CSG medium (2% starch, 1% glucose, 3% sea salt, 0.4% casamino acid, and 0.1% antifoam) was used for final fermentation. The small-scale fermentation was performed in a 250 mL conical flask fitted with a metal spring, 50 mL of CSG medium, and 2% (v/v) inoculation of 3 d TSBY culture, incubating at 30 °C and 220 rpm for 5 d. The large-scale fermentation was performed by using 500 mL conical flask, 150 mL CSG medium and 5% (v/v) inoculation of 3 d TSBY culture, and then incubated at 30 °C and 220 rpm for 7 d. Plasmids used in this study are listed in Table S2.

### Analytical methods

For routine HPLC–MS analysis, 7 mL of fermentation broth was extracted with an equal volume of ethyl acetate and then 5 mL organic phase was collected and dried via nitrogen blowing. The dryness was redissolved in 800 µL methanol and centrifuged at 12,000 rpm for 5 min before injecting 20 µL supernatant for HPLC–MS analysis. The HPLC–MS analysis was conducted on a Waters HPLC coupled with a Waters Acquity QDa detector. For fermentation analysis, a Xbridge C18 column (250 mm × 4.6 mm, 5 μm) was eluted with the mobile phase of acetonitrile and H_2_O (0.1% formic acid, v/v) at a flow rate of 0.8 mL/min in the program of 30–50% for 10 min and then 95% for 12 min. The mass spectrometer ran in positive ionization mode scanning from *m*/*z* 200 to 1250. For HRMS analysis, a Waters XeVO G2-XS Q TOF mass spectrometer was conducted in positive ionization mode, scanning from *m*/*z* 100 to 1200. NMR spectra were recorded on Bruker 600 MHz in DMSO-*d*_6_. Chemical shifts (*δ*) were obtained in reference to tetramethylsilane (TMS) at 0.00 ppm.

### Purification of compounds 1 and 2

To prepare compounds **1** and **2** for structural elucidation, 24 L fermentation broth of LHW50302 was extracted three times with equal volume of ethyl acetate (0.1% formic acid, v/v). The combined organic phases were degreased with hexane and concentrated under vacuum to yield 15 g syrup extract. The extract was subjected to a vacuum liquid chromatography column loaded with silica gel (200–300 mesh) using a stepwise elution with CH_2_Cl_2_/MeOH (1:1, v/v) to afford five fractions (Fr. B1-B5). Guided by HPLC–MS analysis, the B3 fraction (1 g) was selected for further separation by using medium-pressure preparative liquid chromatography (MPLC) equipped with an ODS chromatography column (Santai Technologies, Inc., Spherical C18, 20–45 μm, 100 Å) in an elution condition of 15 mL/min and 10–100% MeOH/H_2_O (0.1% formic acid, v/v) for 3 h to afford sixteen subfractions (Fr. B3A-B3P). Then the fraction B3K (250 mg) was further separated over HPLC (Waters Xbridge C18, 10 × 250 mm, 3.0 mL/min, 35% MeOH/H_2_O) to to yield 2 mg of **1a**, 4 mg of **1b** as well as B3L (33 mg) was purified with HPLC (Waters Xbridge C18, 10 × 250 mm, 3.0 mL/min, 29% CH_3_CN/H_2_O) to obtain 1 mg of **2a** and 2 mg of **2b**.

### Deletion of the ***bpdE***-C gene cassette

To generate the platform mutant strain RJ46 (Δ*bpd*E-C), the *bpd*E-C gene cassette (Fig. 4a) was deleted from the chromosome of LHW50302. To construct the plasmid for introducing double-crossover homologous recombination, a 1227 bp left arm and a 1412 bp right arm were amplified from the genomic DNA of LHW50302, respectively (see primers in Table S1). By using Gibson assembly, the two PCR fragments were assembled with the *E. coli*–*Streptomyces* shuttle plasmid pYH7 [32] linearized by HindIII and NdeI. The resultant pRJ205 plasmid was introduced into LHW50302 strain by conjugation via the transitional host *E. coli* ET12567 containing helper plasmid pUZ8002. The target mutant strains were screened by colony PCR from the apramycin-sensitive colonies prepared after two rounds of propagation on no antibiotics SFM plates. Finally, the mutant strain was reconfirmed by sequencing the PCR products.

### Sit-directed mutagenetic analysis of ***bpdC***, ***bpdD***, and ***bpdE***

The RJ46 mutant was used as a platform host to evaluate the effects of gene mutations within *bpd*E-C cassette by using the mutated *bpd*E-C gene cassette for complementation of RJ46. To this end, the *bpd*E-C gene cassette amplified from LHW50302 was assembled with an integrative vector pIB139 [33] at NdeI and EcoRI sites by using Gibson assembly yielding plasmid pRJ358. Due to failed complementation by using pRJ358 in RJ46 strain, the transcription cassette of *orf*3 (upstream the *bpd*E-C gene cassette) amplified from LHW50302 was introduced into pRJ358 at NsiI and NdeI sites by using Gibson assembly. The resulting plasmid pRJ453 was used as starting material for generating mutations in the *bpd*E-C gene cassette such as the sit-directed mutations in *bpd*E (C89S, C89A, D116A, Y204F, or H245N), *bpd*C (S852A or R792A) and *bpd*D (S551A) and the gene deletion mutation in *bpd*4 (see primers in Table S1). The resulting plasmid (Table S2) was introduced into the RJ46 via conjugation as described above. The target strains were confirmed by sequencing the PCR products.

### Gene deletion and overexpression of ***bpd4***

The *bpd*4 gene deletion mutation was generated based on the complementary plasmid pRJ453. To this end, pRJ453 was cut open with BsrGI and SfiI and then repaired with a partial fragment of *bpd*D (see primers in Table S1) by using Gibson assembly yielding pRJ510. To constructing the *bpd*4 gene overexpression cassettes, the PCR fragment of *bpd*4 was assembled with the fragment of promoter *kasO*p* amplified from pRJ252 [34] at the sites of NsiI and EcoRI in pIB139 (see primers in Table S1). The resulting plasmids pRJ510 and pRJ551 were introduced into RJ46 and LHW50302 by conjugation, respectively.

### Gene deletion of the ***bpd1***

To delete *bpd*1 gene, two PCR fragments, 1411 bp left arm and 1419 bp right arm, were amplified from LHW50302 for homologous recombination (see primers in Table S1). The PCR products were assembled with pYH7 (HindIII, NdeI) by Gibson assembly. The resulting plasmid pRJ509 was introduced into LHW50302 via conjugation. The mutant strain Δ*bpd*1 was screened out by using same strategy described as above.

### Protein structure modeling and ligand docking analysis

The BpdE structure modeled by AlphaFold2 was submitted to Dali server [35] for structural similarity analysis. The ligand docking analysis of BpdE was performed by using Autodock. The structure complex of protein-ligand was read and analyzed by PyMOL2.

### Electronic supplementary material

Below is the link to the electronic supplementary material.

Additional file: Table S1 to S4 and Fig. S1 to S7

## Data Availability

All datasets were generated or analysed during the current study.
