# Supplementary material for: Mutagenetic analysis of the biosynthetic pathway of tetramate bripiodionen bearing 3-(2H-pyran-2-ylidene)pyrrolidine-2,4-dione skeleton
Source: Microb Cell Fact. 2024 Mar 21;23:87. doi: 10.1186/s12934-024-02364-7 (PMC10956176; doi:10.1186/s12934-024-02364-7)

Supporting Information

Mutagenetic analysis of the biosynthetic pathway of tetramate bripiodionen bearing 3-(2*H*-pyran-2-ylidene)pyrrolidine-2,4-dione skeleton

Supplementary Tables

Table S1. Primers used in this study
Table S2. Plasmids used in this work
Table S3. Comparison of experimental and reported ^{1}H and ^{13}C NMR data of **1** (related to Figure S6)
Table S4. ^{1}H and ^{13}C NMR data for **2** in DMSO-d_6 (related to Figure S7)

Supplementary Figs

Fig. S1. Superimpose analysis of the protein structures of BpdE and NcmC
Fig. S2. Multisequence alignment of BpdE homologs
Fig. S3. Multisequence alignment of the A and T domains from BpdC and BpdD
Fig. S4. HPLC–MS analysis of Δ*bpd*1 and multisequence alignment of Bpd1
Fig. S5. The natural products and related BGCs containing both discrete A-T didomain and A-less module
Fig. S6. NMR spectra of **1** (related to Table S3)
Fig. S7. NMR spectra of **2** (related to Table S4)

Table S1. Primers used in this study.

Primers	Sequence (5' to 3')	Descriptions
**RJ46 (Δ*bpd*E-C) mutant generation, deletion of the *bpd*E-C gene cassette in the chromosome of LHW50302**		
L-bpdEDC-S	ACTGATCAAGGCGAATACTTCAAGGACTTCGCGCGTCTTTTCTTAC (22 bp overlapping with the *Nde*I side of pYH7)	1227 bp left arm amplified from LHW50302 for homologous recombination
L-bpdEDC-A	ACAGCTCGGTGGTGCTCTCCTCCTCGTACAGCTCGATCAG (39 bp overlapping with the right arm)	
R-bpdEDC-S	TGATCGAGCTGTACGAGGAGGAGAGCACCACCGAGCTGTTCCTG	1412 bp right arm amplified from LHW50302 for homologous recombination
R-bpdEDC-A	AGATCCGTCGACCTGCAGGCATGCAGTGGTCGGACATCTCTTTC (25 bp overlapping with the *Hind*III side of pYH7)	
T-bpdEDC-S	ACTTCGGCAAGGTCACGGAG	Mutant screening
T-bpdEDC-A	TGACCGGTGTGCACCTCG	
**Clone the *bpd*E-C gene cassette to generate pRJ453**		
EDC-S	AATCGTGCCGGTTGGTAGGATCCACATATGACCGAGGCCGTTAATCCCTG (26 bp overlapping with the *Nde*I side of pIB139)	The 5930 bp fragment of *bpd*E-C gene cassette amplified from LHW50302 was introduced into pIB139 at *Nde*I and *Eco*RI sites yielding pRJ358.
EDC-A	AACAGCTATGACATGATTACGAATTCAGCCGACGGGGCCGGTGACGTG (21 bp overlapping with the *Eco*RI side of pIB139)	
Promoter-orf3-S	TGGGCTGCAGGTCGACTCTAGTATGCAACGGTGCGGGCGAGGGAGTCTC (27 bp overlapping with the *Nsi*I side of pRJ358)	The 875 bp fragment of *orf*3 transcription cassette amplified from LHW50302 was introduced into pRJ358 at *Nsi*I and *Nde*I sites yielding pRJ453.
Promoter-orf3-A	ACCCAGGGATTAACGGCCTCGGTCATCTTGTTTACTCAACTCCCTGTTACTCCAC (25 bp overlapping with the *Nde*I side of pRJ358)	
bdpEDC-T-S	GGTCTCGACGACGACTTCTTC	Colony PCR
bdpEDC-T-A	GACGGTGCTGACGGTGATG	
**Generate *bpd*D (S551A), *bpd*C (S852A), and *bpd*C (R792A) site-mutations based on pRJ453**		
1-bpdD-S	TGCTCGACGCGGAGCTGAACCGGGTG (49 bp overlapping with the *Bsr*GI side of pRJ453)	A 1286 bp fragment containing *bpd*D (S551A) site-mutation was generated by overlapping PCR and the fragment was introduced into pRJ453 at *Bsr*GI and *Sfi*I sites to yield pRJ512.
1-EDC-A	AGGAGGGCGTCCCCGCCCAGCGCGAAG (26 bp overlapping with the second part of the gene)	
2-EDC-S	TTCGCGCTGGGCGGGGACGCCCTCCTG	
2-bpdD-A	TCCGCCGGGCTCCTCGCCGCGCCAG (50 bp overlapping with the *Sfi*I side of pRJ453)	
T-S551A-S	TGGGCCGCATGGACGATCAG	Colony PCR
T-S551A-A	TGGATCGGCAGCACGATCCGTC	
1-bpdC-S	TGGAACGCCGACGGCACCCTGGAGG (43 bp overlapping with the *Asc*I side of pRJ453)	A 1009 bp fragment containing the *bpd*C (R792A) site-mutation was generated by overlapping PCR and the fragment was introduced into pRJ453 at *Asc*I and *Pci*I sites to yield pRJ503.
2-EDC-A	TCGGGGGCCCCGTCCTTGAGCGGCAGTG (19 bp overlapping with the second part of the gene)	
3-EDC-S	TCAAGGACGGGGCCCCCGACCGCGAAC	
2-bpdC-A	ACGGTTCCTGGCCTTTTGCTGGCCTTTTGCTC (33 bp overlapping with the *Pci*I side of pRJ453)	
1-bpdC-S	Same as above	A 1022 bp fragment of *bpd*C (S852A) was generated by overlapping PCR and the fragment was introduced into pRJ453 at *Asc*I and *Pci*I sites to yield pRJ502.
1-PCP2-A	AGCAGGGCGTGGCCGCCCTCGGTGAAGAAG (23 bp overlapping with the second part of the gene)	
2-PCP2-S	ACCGAGGGCGGCCACGCCCTGCTCGGCGCGCAAC	
2-bpdC-A1	ACGCGGCCTTTTTACGGTTCCTGGCCTTTTGCTGGCCTTTTGC (46 bp overlapping with the *Pci*I side of pRJ453)	
T-bpdC-S	TGCGCCGCGTCACCACCGCCGAG	Colony PCR
T-bpdC-A	AGCCGACGGGGCCGGTGACGTG	
**Generate site-mutations in *bpd*E gene based on pRJ453**		
Promoter-orf3-S	TGGGCTGCAGGTCGACTCTAGTATGCAACGGTGCGGGCGAGGGAGTCTC (27 bp overlapping with the *Nsi*I side of pRJ453)	A 1680 bp fragment containing *bpd*E (C89A) mutation was generated by overlapping PCR

1-C89A-A	ACACTGCCCACGGCGTAGCCCAGCACCGTACCG ACC	and the fragment was introduced into pRJ453 at *Nsi*I and *Eco*RV sites to yield pRJ515.
2- C89A-S	TGCTGGGCTACGCCGTGGGCAGTGTCTTCGCCTC (25 bp overlapping with the promoter *native*-p*)	
BpdE-A	TGACGACCTGCCCGGTCACCGCCGATATCACTCC (26 bp overlapping with the *Eco*RV side of pRJ453)	
Promoter-orf3-S	Same as above	A 1680 bp fragment containing the *bpd*E (C89S) mutation was generated by overlapping PCR and the fragment was introduced into pRJ453 at *Nsi*I and *Eco*RV sites to yield pRJ520.
1-C89S-A	ACACTGCCCACGGAGTAGCCCAGCACCGTACCG ACCGGCCTG	
2- C89S-S	TGGGCTGCAGGTCGACTCTAGTATGCAACGGTGC GGGCGAGGGAGTCTC (25 bp overlapping with the promoter *native*-p*)	
BpdE-A	Same as above	
Promoter-orf3-S	Same as above	A 1680 bp fragment containing the *bpd*E (D116A) mutation was generated by overlapping PCR and the fragment was introduced into pRJ453 at *Nsi*I and *Eco*RV sites to yield pRJ516.
1-D116A-A	TGGGCATCTCCGGGGCGAAGAGCAGGATGCGCG GCCTGCTG	
2-D116A-S	ATCCTGCTCTTCGCCCCGGAGATGCCCAACAC (28 bp overlapping with the promoter *native*-p*)	
BpdE-A	Same as above	
Promoter-orf3-S	Same as above	A 1680 bp fragment containing the *bpd*E (Y204F) mutation was generated by overlapping PCR and the fragment was introduced into pRJ453 at *Nsi*I and *Eco*RV sites to yield pRJ518.
1-Y204F-A	TCCGGGCGGCGGCCAGGAAGGACACGTACGCCC TGAAGAGGCCCAG	
2-Y204F-S	TACGTGTCCTTCCTGGCCGCCGCCCGGAAGCTG (28 bp overlapping with the promoter *native*-p*)	
BpdE-A1	TGACGACCTGCCCGGTCACCGCCGATATCACTCC CCCTTC (26 bp overlapping with the *Eco*RV side of pRJ453)	
Promoter-orf3-S	Same as above	A 1680 bp fragment containing the *bpd*E (H245N) mutation was generated by overlapping PCR and the fragment was introduced into pRJ453 at *Nsi*I and *Eco*RV sites to yield pRJ544.
1-H245N-A	AGGAGCTCGTCGTTGCTCGCCTCGAAGCGCAGCT CCTCG	
2- H245N-S	TGCGCTTCGAGGCGAGCAACGACGAGCTCCTGC GCTC (31 bp overlapping with the promoter *native*-p*)	
BpdE-A1	Same as above	
T-promoter-S<br>T-promotor-A	TCAGGCGCCATTCGCCATTC<br>AGGCTCAGCGTGTTGGGCATC	Colony PCR
**Generate *bpd*1 gene deletion in the chromosome of LHW50302**		
L-bpd1-S	ACTGATCAAGGCGAATACTTCACGGGTCTTCGTG CTGGTGATC (22 bp overlapping with the *Nde*I side of pYH7)	The 1411 bp left arm was amplified from LHW50302 for homologous recombination.
L-bpd1-A	TGGTATGCGCACGGGCGACGGAGTGGTAGTAG (22 bp overlapping with the right arm)	
R-bpd1-S	TCCGTCGCCCGTGCGCATACCAGGCGGGACGCG GAAG	The 1419 bp right arm was amplified from LHW50302 for homologous recombination.
R-bpd1-A	AGATCCGTCGACCTGCAGGCATGCAAAAAGACG CGCGAAGTCCTG (25 bp overlapping with the *Hind*III side of pYH7)	
T-509-S<br>T-509-A	ACCCCGTCCCCCCACTCCGCAC<br>TCGTGCCGGTCGCGCATGTC	Mutant screening
**Generate *bpd*4 gene deletion based on pRJ453**		
bpdD-S	AACCGGGTGGAGCCGGGGCTCACAGGGGAGTTGT ACATCGCCGGGGAG (32 bp overlapping with the *Bsr*GI side of pRJ453)	The pRJ453 was digested by *Bsr*GI and *Sfi*I and then a 847 bp fragment amplified from pRJ453 was introduced into the sites to yield pRJ510.
bpdD-A	GGCGCCGGCAGGTCGGCCCGCGGGATCGGCAGCA CGATCCGTCCTGTGCCGGAAG (22 bp overlapping with the *Sfi*I side of pRJ453)	
Tbpd4-S<br>Tbpd4-A	AACGGCAAGCTCGACACCAAG<br>TCATCAGGACAGCTCTCCGTTTC	Colony PCR
**Construct *bpd*4 gene overexpression cassette based on pIB139**		
1-KasOp-orf4-S	TGGGCTGCAGGTCGACTCTAGTATGCATTGTTCA CATTCGAACGGTC (27 bp overlapping with the *Nsi*I side of pIB139)	The 591 bp fragment of *bpd*4 gene amplified from pRJ453 and the 139 bp fragment of *KasO*p* promoter amplified from the pRJ252 were assembled into the
1-KasOp-orf4-A	TGGATCGGCAGCACAACTCCCCCAGTCCTGCAC	
2-KasOp-orf4-S	ACTGGGGGAGTTGTGCTGCCGATCCACGCGCGT	

2-KasOp-orf4-A	GGAC<br>(26 bp overlapping with the promoter *KasO*p*)<br>AACAGCTATGACATGATTACGAATTCAGGACAG<br>CTCTCCGTTTCCGCGGGCCAC<br>(21 bp overlapping with the *Eco*RI side of pIB139)	*Nsi*I and *Eco*RI sites of pIB139 to yield pRJ551.
T-orf4-S<br>pIB139-A	TGTAAAACGACGGCCAGT<br>TGAGTTAGCTCACTCATTAGGCAC	Colony PCR

Table S2. Plasmids used in this work.

Plasmids	Resistance	Descriptions
pRJ205	aprR, ampR	to generate *bpd*E-C deletion
pRJ509	aprR, ampR	to generate *bpd*1 in-frame deletion
pRJ453	aprR	cloning of *bpd*E-C gene cassette based on the integrative vector pIB139
pRJ502	aprR	pRJ453 derivative, *bpd*C (S852A)
pRJ503	aprR	pRJ453 derivative, *bpd*D (R792A)
pRJ512	aprR	pRJ453 derivative, *bpd*D (S551A)
pRJ510	aprR	pRJ453 derivative, *bpd*4 gene deletion
pRJ515	aprR	pRJ453 derivative, *bpd*E (C89A)
pRJ516	aprR	pRJ453 derivative, *bpd*E (D116A)
pRJ518	aprR	pRJ453 derivative, *bpd*E (Y204F)
pRJ520	aprR	pRJ453 derivative, *bpd*E (C89S)
pRJ544	aprR	pRJ453 derivative, *bpd*E (H245N)
pRJ551	aprR	cloning of *bpd*4 under *KasO*p* promoter based on the pIB139
Abbreviation: amp, ampicillin; apr, apramycin		

Table S3. ^{1}H (600 MHz) and ^{13}C (150 MHz) NMR data for **1** (**1a** (*E*-$\Delta^{3(5)}$) and **1b** (*Z*-$\Delta^{3(5)}$)) in DMSO-d_6.

No.	1a		1b		Bripiodionen (5*E*-form) [a]		Bripiodionen (5*Z*-form) [a]	
	δ_C, type	δ_H, mult (*J* in Hz)	δ_C, type	δ_H, mult (*J* in Hz)	δ_C, type	δ_H, mult (*J* in Hz)	δ_C, type	δ_H, mult (*J* in Hz)
**1**	57.3, CH	3.90, dd (4.5, 7.5)	56.7, CH	3.90, dd (4.5, 7.5)	57.4, CH	3.91, dd (4.2, 7.3)	56.8, CH	3.89, dd (4.3, 7.4)
**1-NH**		7.69, s		7.94, s		7.70, s		7.94, s
**2**	199.1, C		196.0, C		199.3, C		196.2, C	
**3**	102.4, C		102.4, C		102.5, C		102.5, C	
**4**	168.4, C		169.0, C		167.5, C		169.1, C	
**5**	167.4, C		165.4, C		166.9, C		165.4, C	
**6**	118.5, CH	7.46, d (9.8)	118.2, CH	7.63, d (9.8)	118.6, CH	7.45, d (9.8)	118.3, CH	7.61, d (9.8)
**7**	150.4, CH	7.06, dd (6.7, 9.9)	149.8, CH	7.04, dd (6.7, 9.8)	150.5, CH	7.04, dd (9.8, 6.7)	149.9, CH	7.03, dd (9.8, 6.7)
**8**	29.5, CH	2.66, m	29.6, CH	2.66, m	29.6, CH	2.64, m	29.6, CH	2.64, m
**9**	84.3, CH	3.76, dd (3.2, 10.6)	84.4, CH	3.76, dd (3.2, 10.6)	84.4, CH	3.74, dd (3.1, 10.4)	84.4, CH	3.74, dd (3.1, 10.4)
**10**	28.8, CH	1.90, m	28.8, CH	1.90, m	28.9, CH	1.89, m	28.9, CH	1.89, m
**11**	17.5, CH_3	0.89, d (6.5)	17.4, CH_3	0.89, d (6.5)	17.6, CH_3	0.87, d (4.7)	17.6, CH_3	0.87, d (4.7)
**12**	37.8, CH_2	2.28, dd (7.3, 15.5)<br>2.46, dd (4.5, 15.5)	37.7, CH_2	2.24, dd (7.4, 15.4)<br>2.46, dd (4.5, 15.4)	37.9, CH_2	2.27, dd (7.3, 15.5)<br>2.46, dd (4.2, 15.5)	37.8, CH_2	2.24, dd (7.4, 15.5)<br>2.45, dd (4.3, 15.5)
**13**	170.9, C		171.0, C		171.1, C		171.1, C	
**13-NH_2**		6.87, s<br>7.36, s		6.87, s<br>7.36, s		6.88, s<br>7.36, s		6.88, s<br>7.36, s
**14**	10.4, CH_3	0.90, d (7.2)	10.5, CH_3	0.90, d (7.2)	10.5, CH_3	0.88, d (4.9)	10.5, CH_3	0.88, d (4.9)
**15**	18.8, CH_3	1.17, d (7.2)	19.2, CH_3	1.18, d (6.7)	19.3, CH_3	1.15, d (6.4)	19.3, CH_3	1.15, d (6.4)

[a] *J Nat Prod*. 1997;60(5):529-532.

Table S4. ^{1}H (600 MHz) and ^{13}C (150 MHz) NMR data for **2** (**2a** (E-$\Delta^{3(5)}$) and **2b** (Z-$\Delta^{3(5)}$)) in DMSO-d_6.

No.	**2a**				**2b**	
	δ_C, type	δ_H, mult (J in Hz)	COSY	HMBC	δ_C, type	δ_H, mult (J in Hz)
**1**	57.3, CH	3.92, dd (4.1, 7.3)	12		56.7, CH	3.89, dd (4.5, 7.3)
**1-NH**		7.65, s		1, 2, 3		7.93, s
**2**	199.2, C				196.0, C	
**3**	102.4, C				102.4, C	
**4**	167.5, C				169.0, C	
**5**	166.9, C				165.4, C	
**6**	118.6, CH	7.46, d (9.8)	7	5, 8	118.3, CH	7.62, d (9.8)
**7**	150.6, CH	7.06, dd (6.6, 9.8)	6, 8		150.0, CH	7.05, dd (6.6, 9.8)
**8**	29.6, CH	2.64, m	7, 14		29.6, CH	2.64, m
**9**	82.5, CH	3.85, dd (3.1, 10.6)	10		82.5, CH	3.85, dd (3.1, 10.6)
**10**	34.6, CH	1.72, m	9, 11		34.7, CH	1.72, m
**11**	10.4, CH_3	0.85, d (6.8)	10	15	10.4, CH_3	0.85, d (6.8)
**12**	37.9, CH_2	2.27, dd (7.3, 15.4)<br>2.45, dd (4.6, 15.6)	1	13	37.7, CH_2	2.25, dd (7.3, 15.4)<br>2.45, dd (4.6, 15.5)
**13**	171.0, C				171.0, C	
**13-NH_2**		6.87, s<br>7.36, s				6.87, s<br>7.36, s
**14**	10.4, CH_3	0.90, d (4.8)	8	7, 9	10.4, CH_3	0.90, d (4.8)
**15**	23.8, CH_2	2.14, m	16		23.9, CH_2	2.14, m
**16**	13.4, CH_3	0.88, t (7.4)	15	10	13.1, CH_3	0.88, t (7.4)

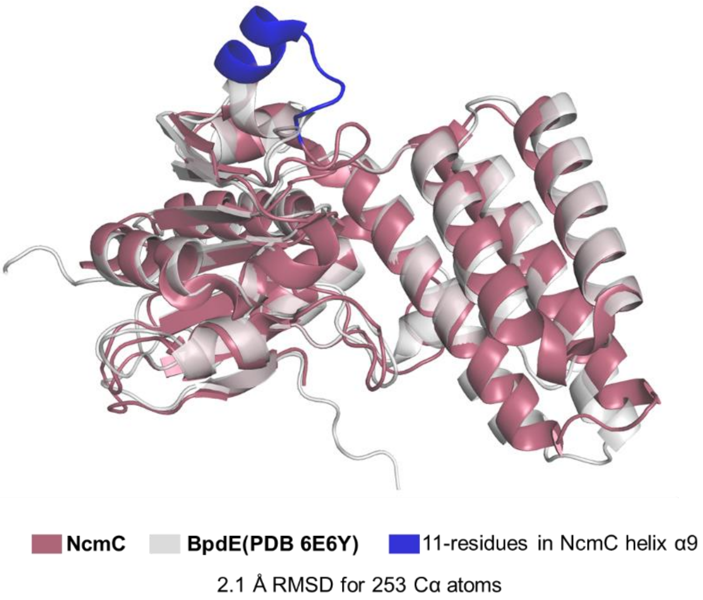

Fig. S1. Superimpose analysis of the protein structures of BpdE and NcmC

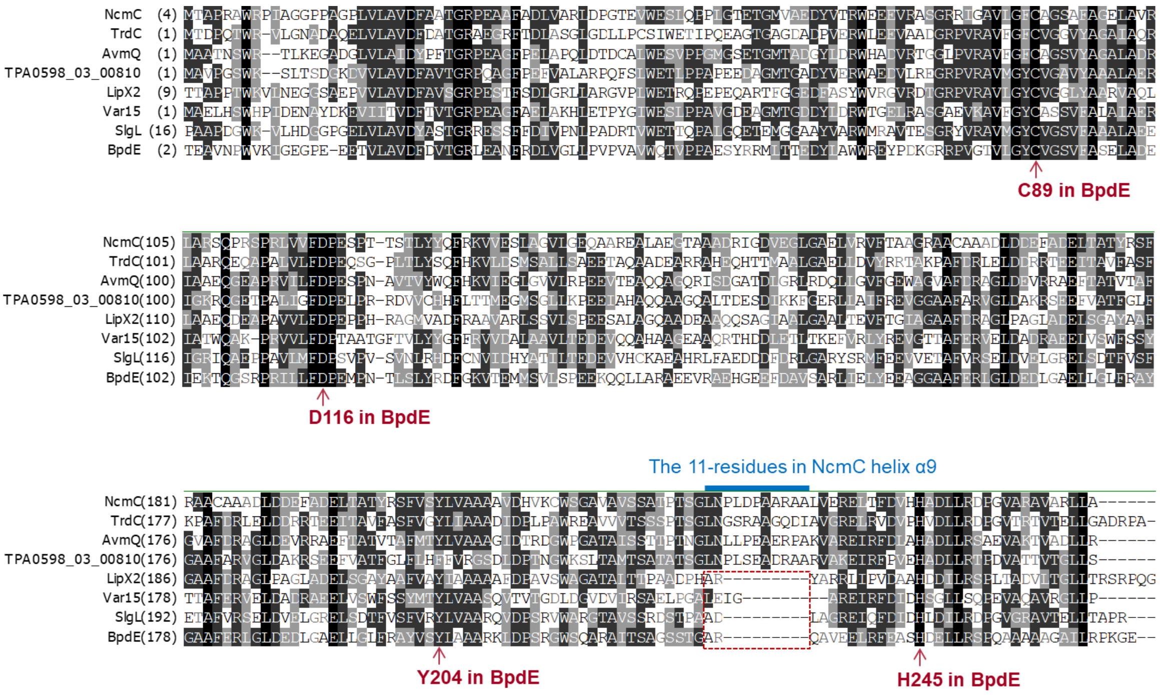

Fig. S2. Multisequence alignment of BpdE homologs

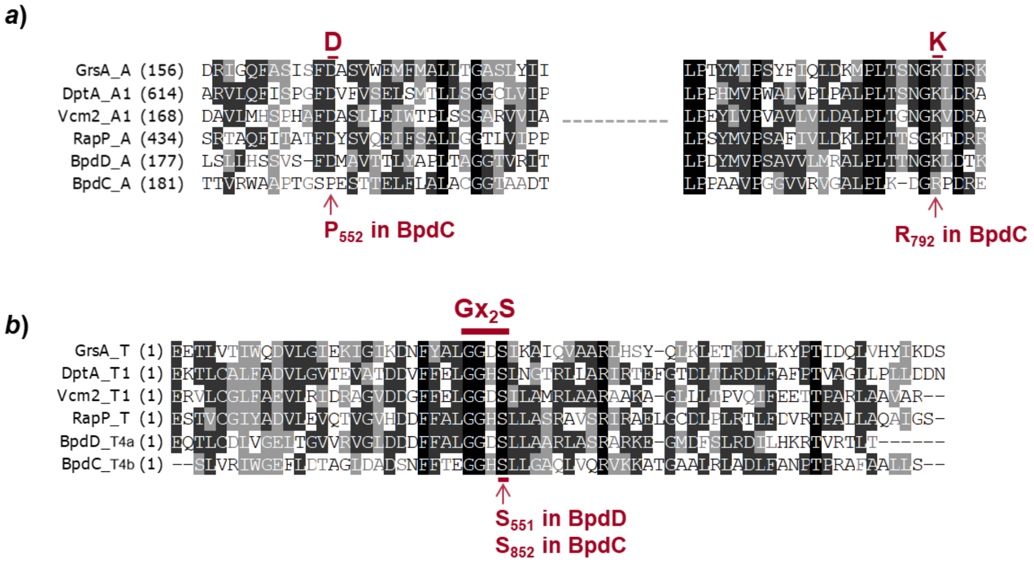

UniProtKB No: GrsA: KON97029.1, **DptA**: AAX31557.1, **Vcm2**: AEI58865.1, **RapP**: CAA60461.1

Fig. S3. Multisequence alignment of the A and T domains from BpdC and BpdD

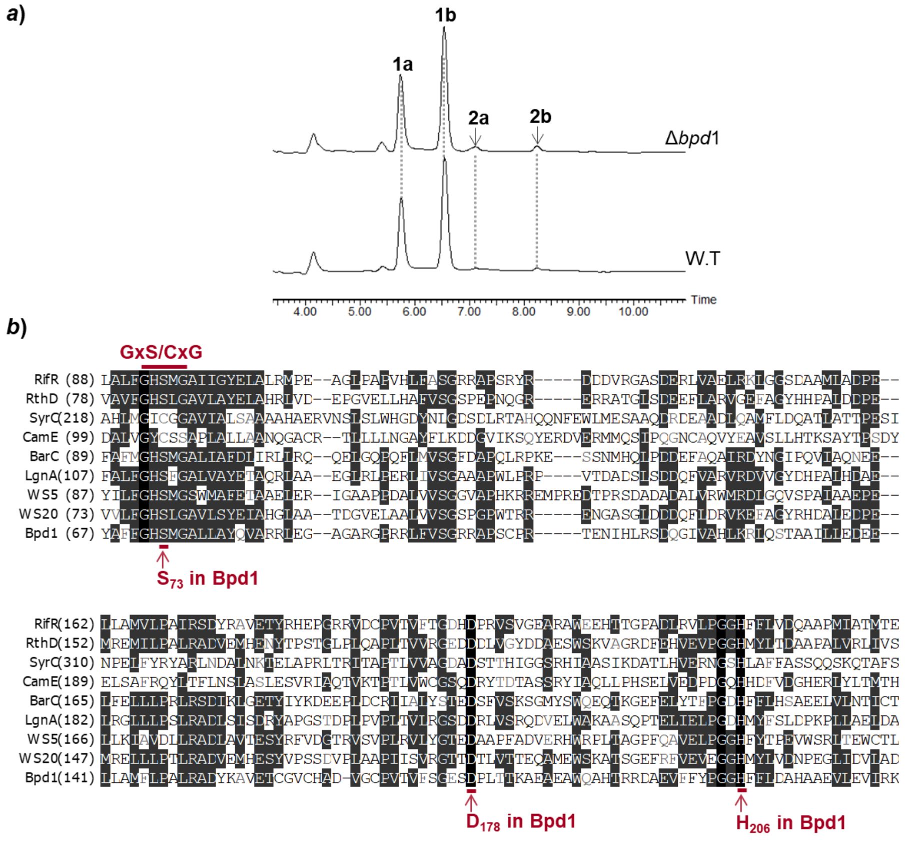

Fig. S4. HPLC–MS analysis of Δ*bpd*1 and multisequence alignment of Bpd1

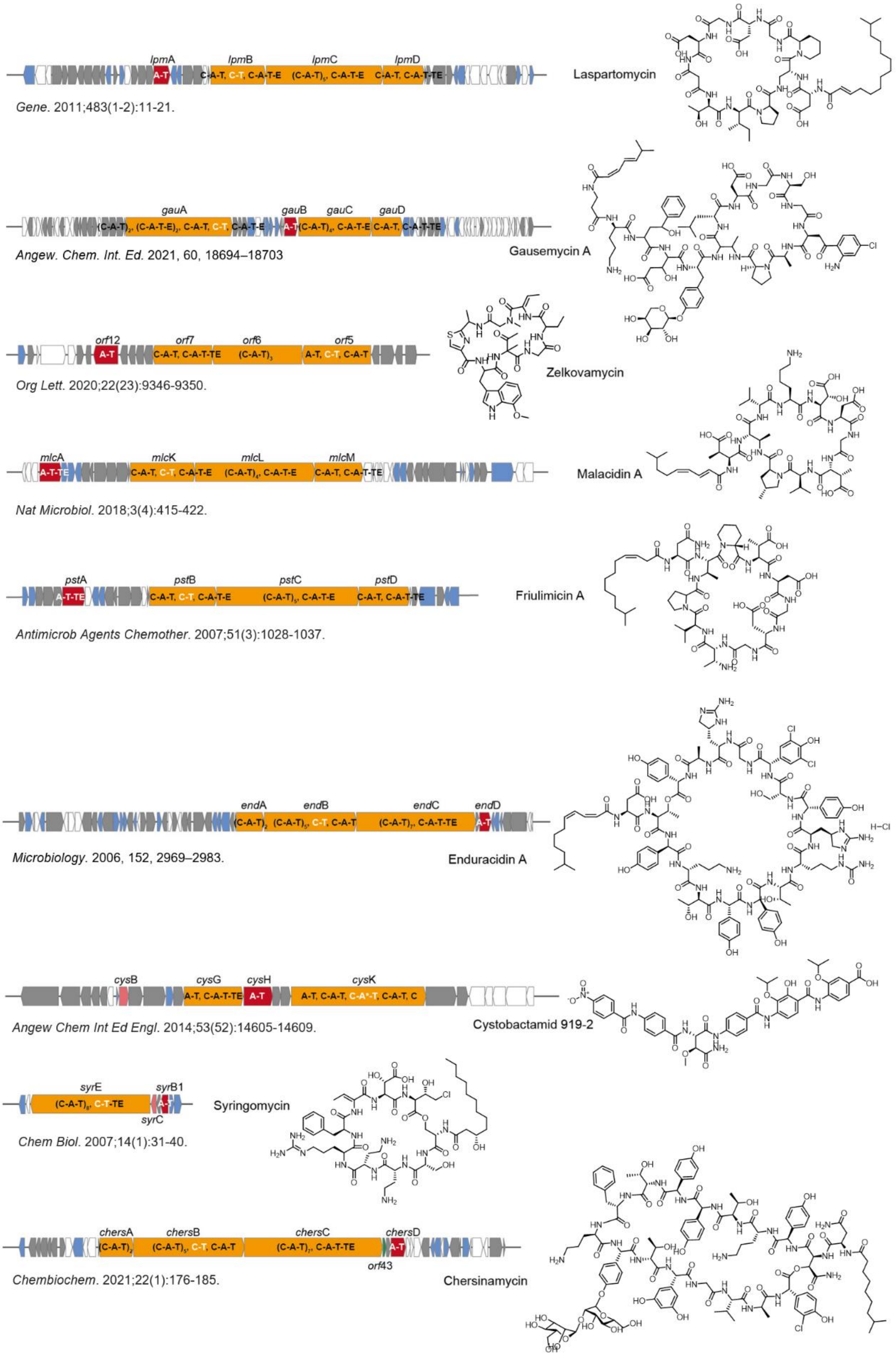

lpmA
lpmB
lpmC
lpmD
Gene. 2011;483(1-2):11-21.
Laspartomycin
gauA
gauB
gauC
gauD
Angew. Chem. Int. Ed. 2021, 60, 18694–18703
Gausemycin A
orf12
orf7
orf6
orf5
Org Lett. 2020;22(23):9346-9350.
Zelkovamycin
mlcA
mlcK
mlcL
mlcM
Nat Microbiol. 2018;3(4):415-422.
Malacidin A
pstA
pstB
pstC
pstD
Antimicrob Agents Chemother. 2007;51(3):1028-1037.
Friulimicin A
endA
endB
endC
endD
Microbiology. 2006, 152, 2969–2983.
Enduracidin A
cysB
cysG
cysH
cysK
Angew Chem Int Ed Engl. 2014;53(52):14605-14609.
Cystobactamid 919-2
syrE
syrB1
syrC
Chem Biol. 2007;14(1):31-40.
Syringomycin
chersA
chersB
chersC
chersD
orf43
Chembiochem. 2021;22(1):176-185.
Chersinamycin

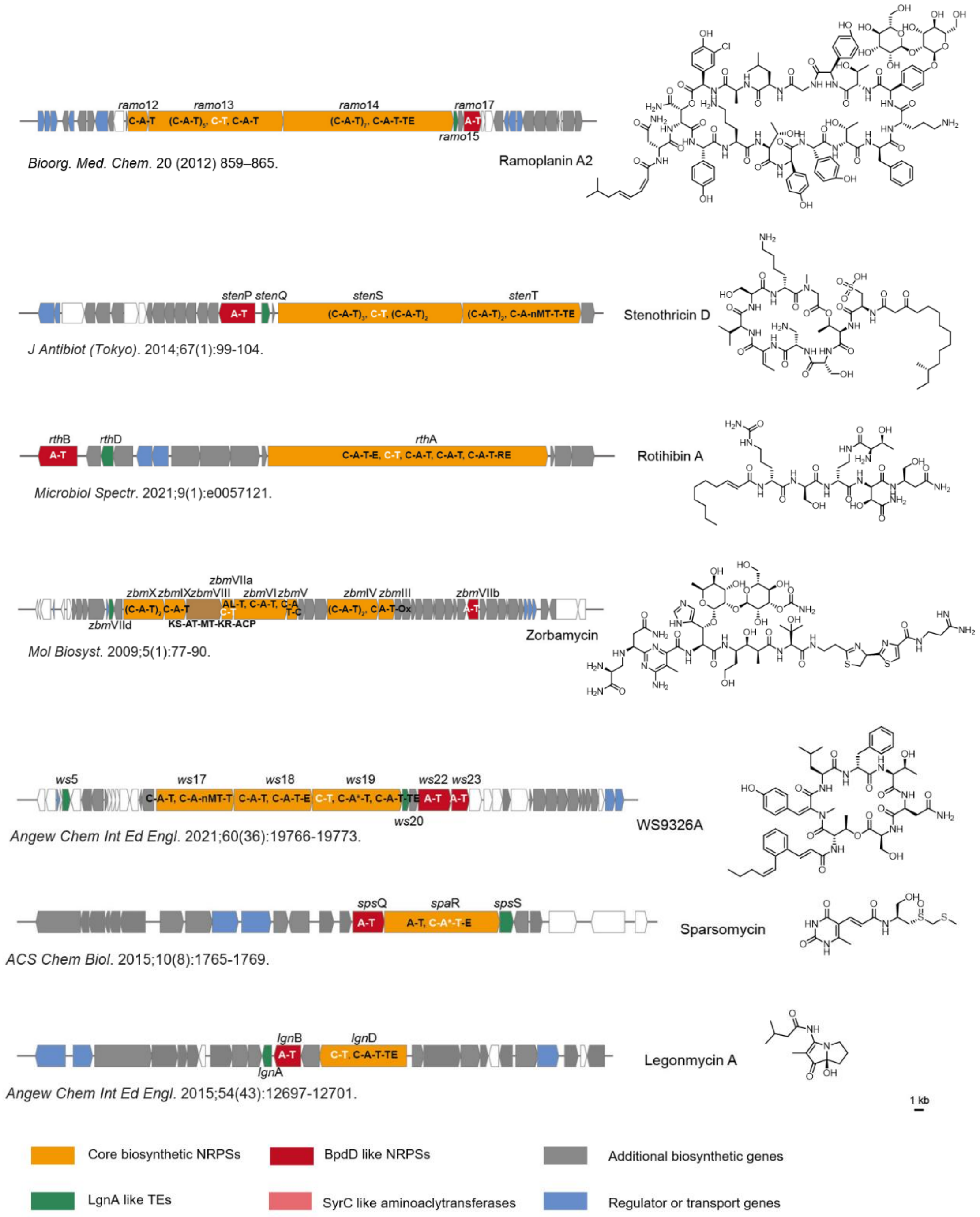

Fig. S5. The natural products and related BGCs containing both discrete A-T didomain and A-less module

Fig. S6. NMR spectra of compound **1** (related to Table S3).

a. ^{1}H-NMR spectrum of **1**.

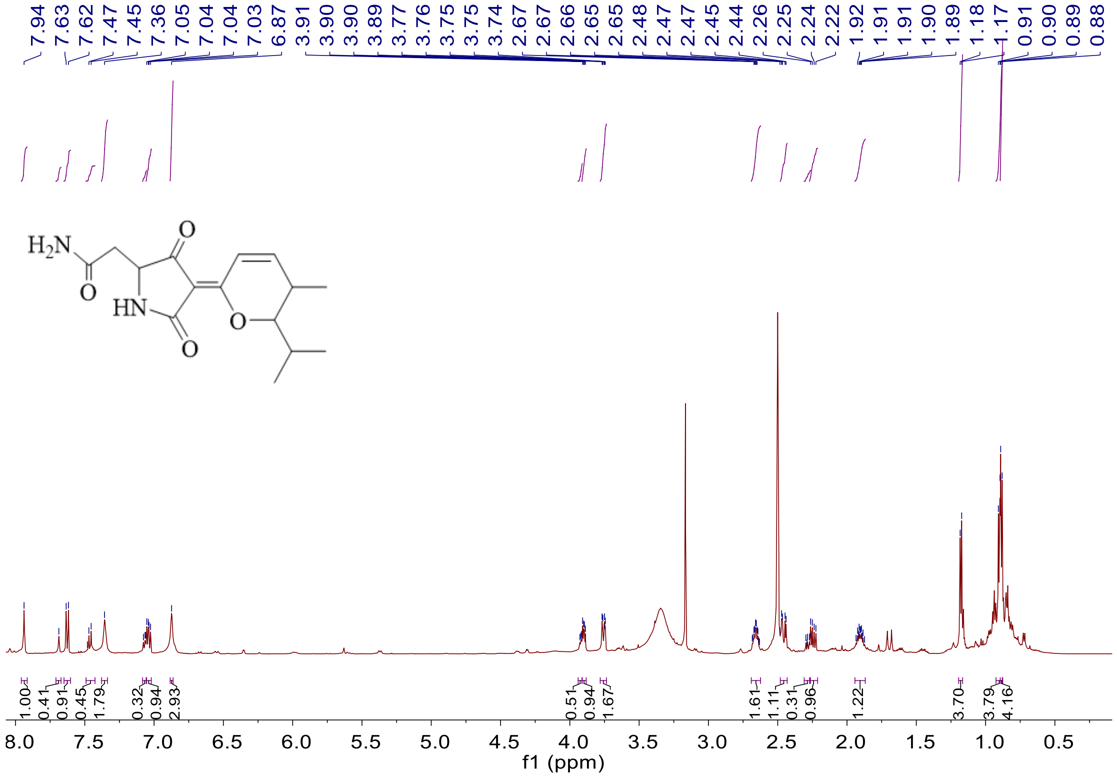

b. ^{13}C-NMR spectrum of **1**.

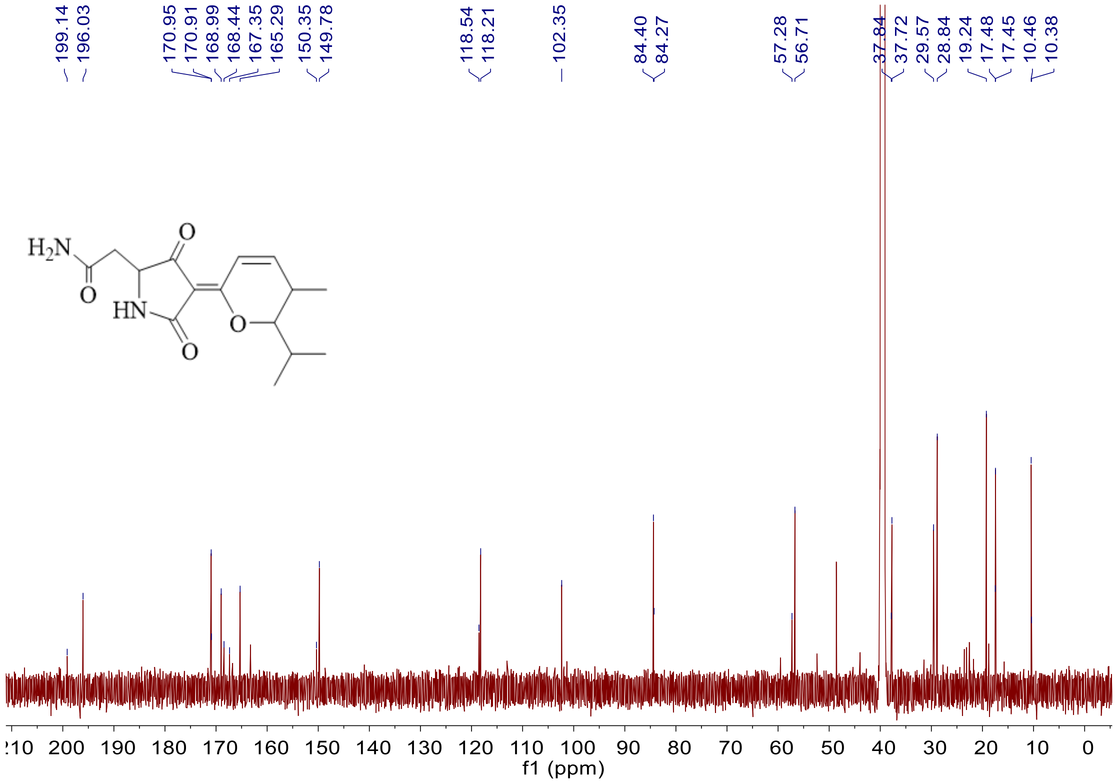

Fig. S7. NMR spectra of compound **2** (related to Table S4).

a. ^{1}H-NMR spectrum of **2**.

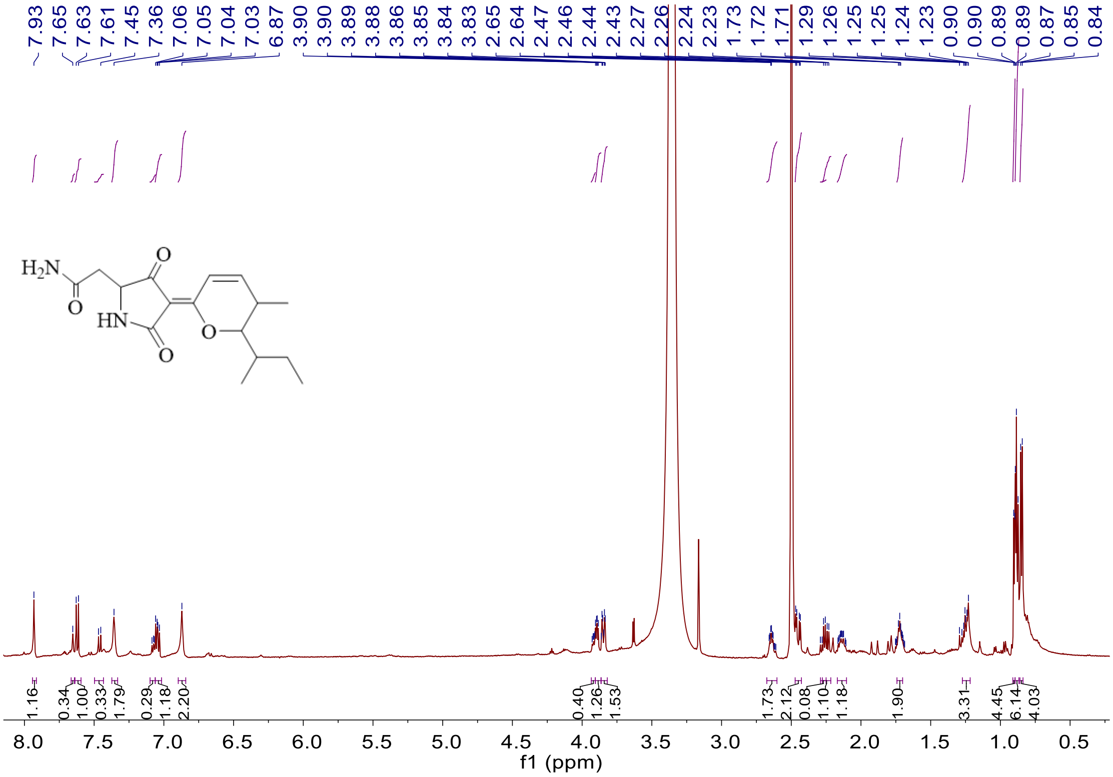

b. ^{13}C-NMR spectrum of **2**.

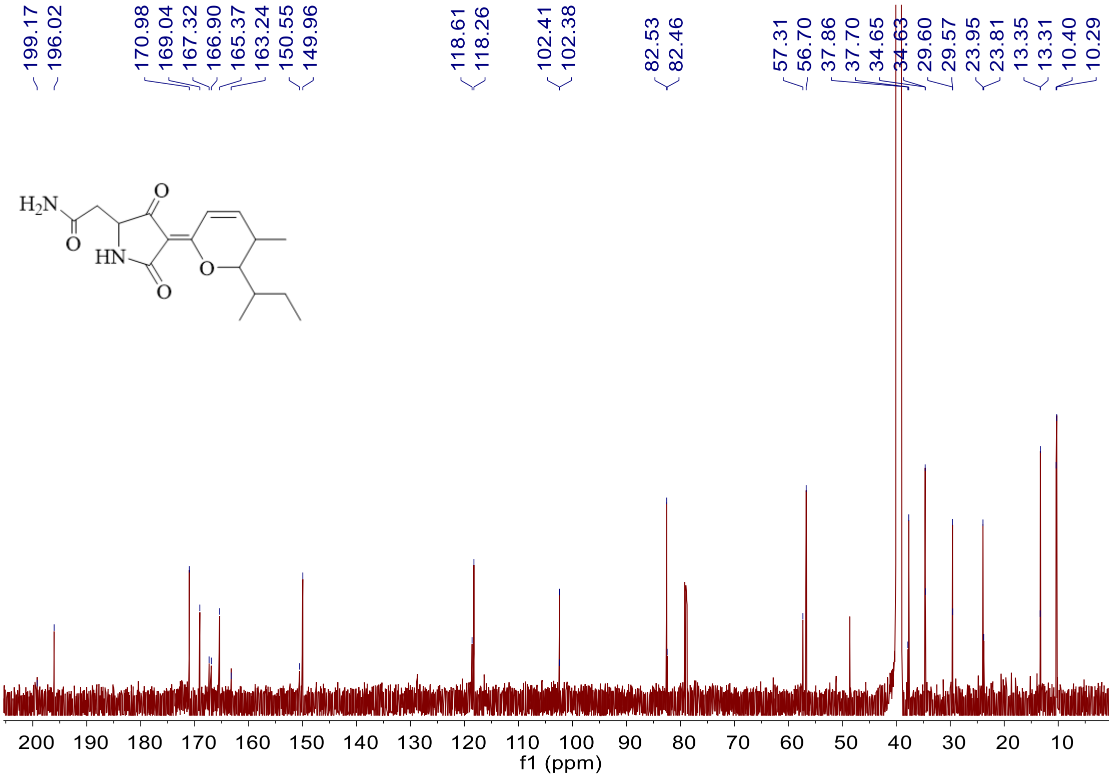

c. DEPT-NMR spectrum of **2**.

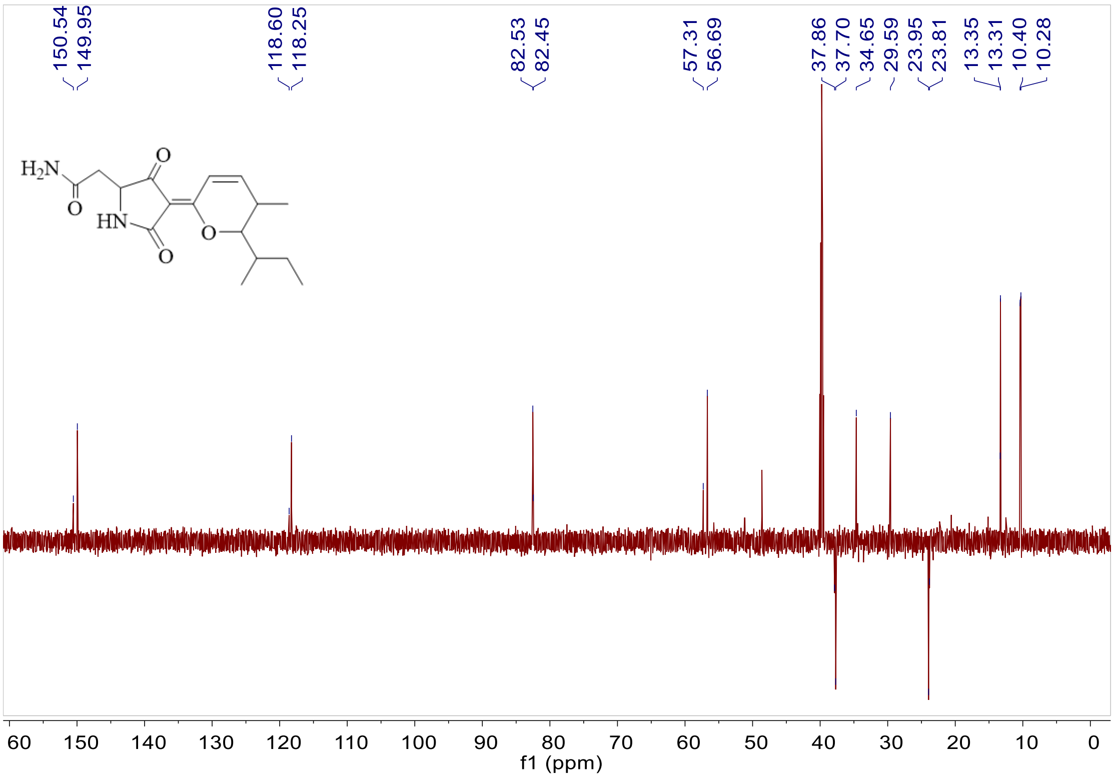

d. COSY spectrum of **2.**

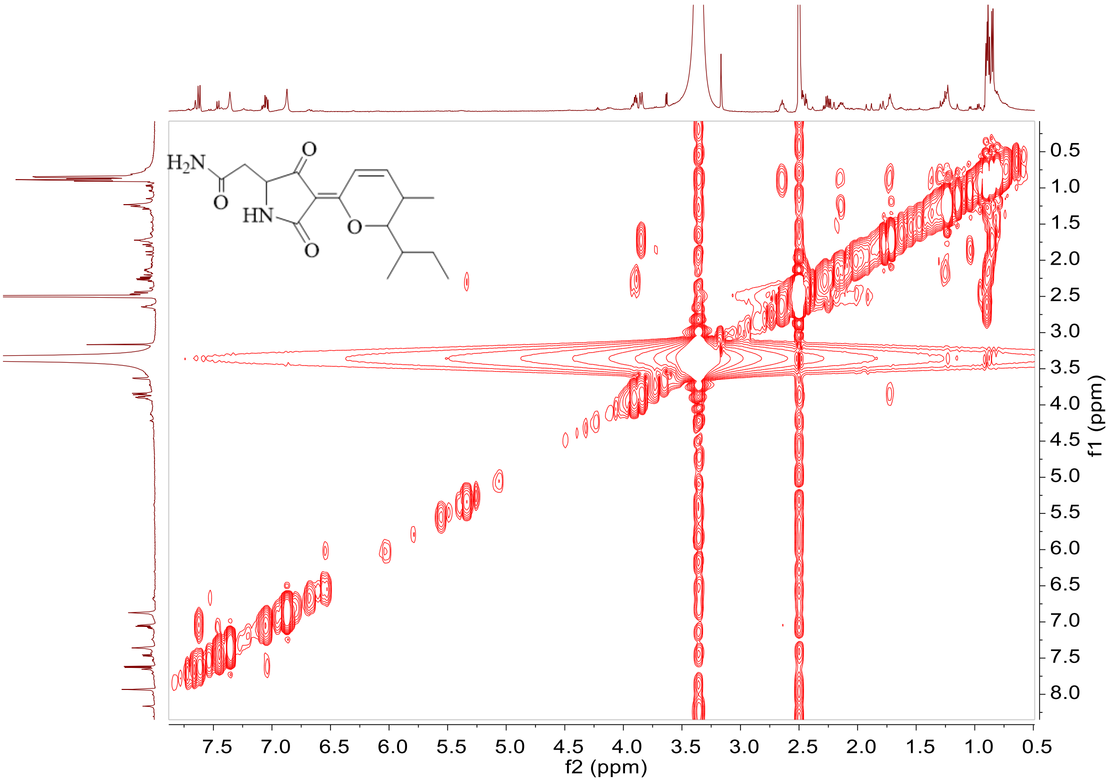

e. HSQC spectrum of **2**.

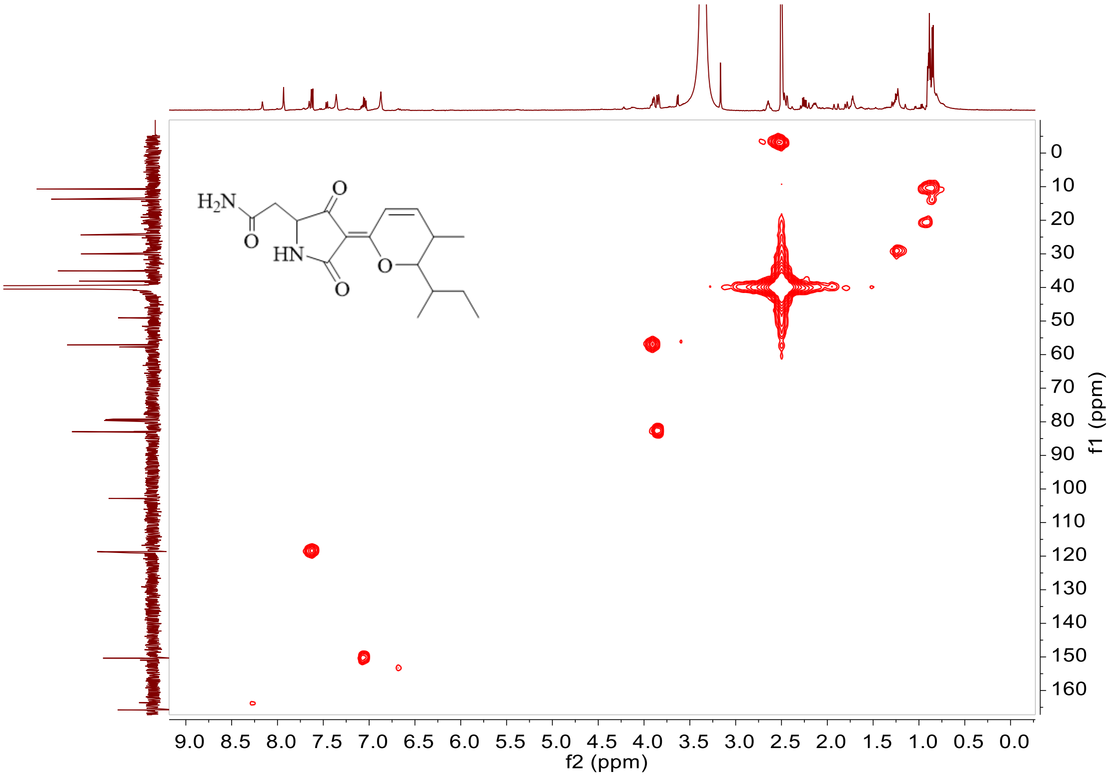

f. HMBC spectrum of **2**.

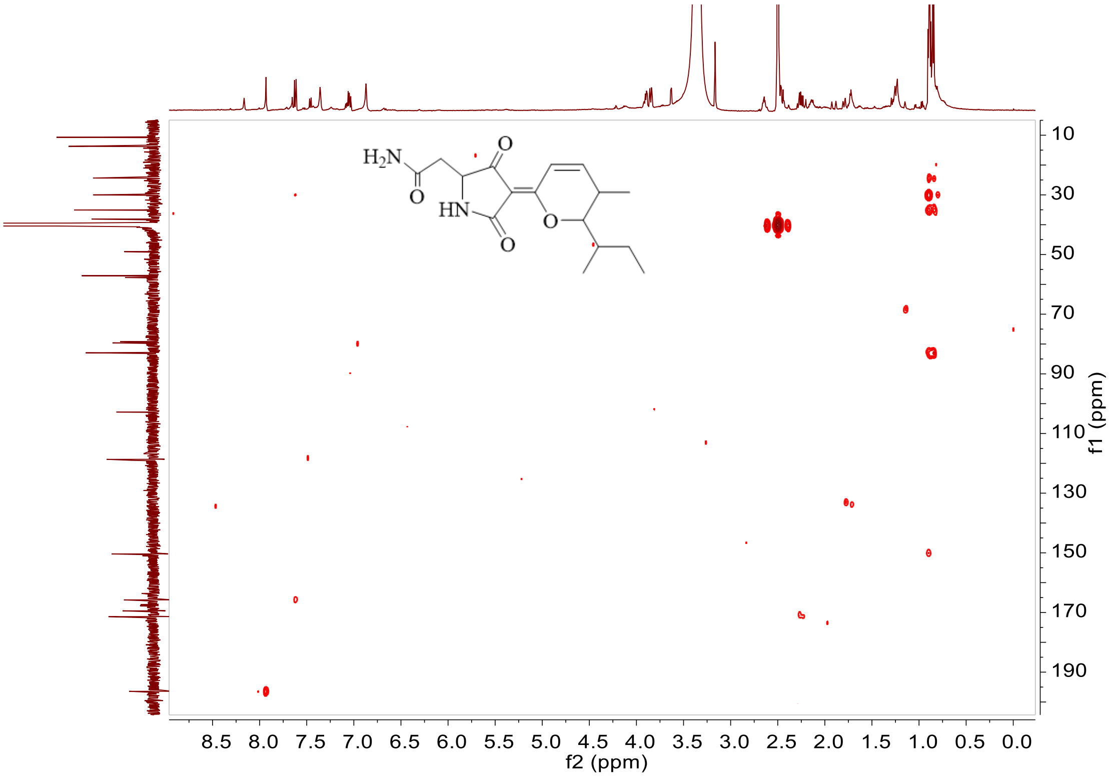

g. NOESY spectrum of **2**.

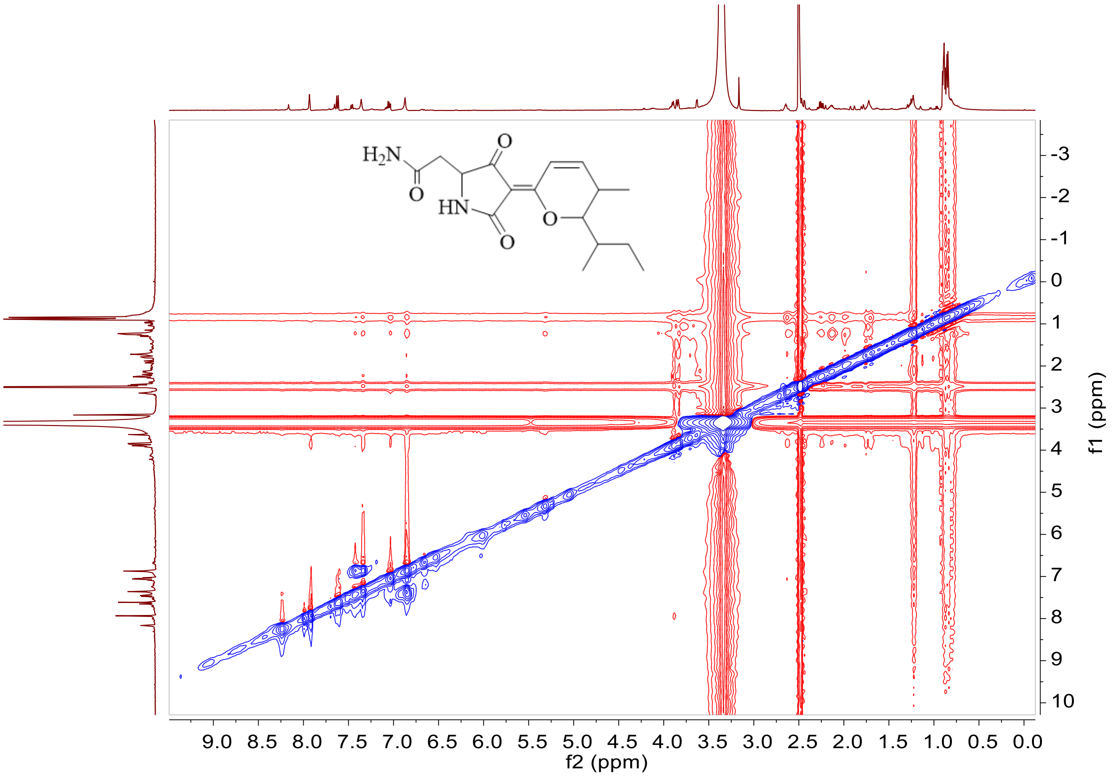

Supplement: Supplementary file 1 — Additional file: Table S1 to S4 and Fig. S1 to S7 [file 12934_2024_2364_MOESM1_ESM.pdf]
